# Decoding cardiac metabolic reprogramming through single-cell multi-omics: from mechanisms to therapeutic applications

**DOI:** 10.3389/fcell.2025.1710474

**Published:** 2025-12-01

**Authors:** Wanqi Rong, Yabin Zhou

**Affiliations:** 1 First Clinical Medical School, Heilongjiang University of Chinese Medicine, Harbin, China; 2 Department of Cardiology, The First Hospital of Heilongjiang University of Chinese Medicine, Harbin, China

**Keywords:** cardiac metabolic reprogramming, single-cell multi-omics, cellular heterogeneity, precision medicine, interdisciplinary collaboration

## Abstract

Cardiac metabolic reprogramming is a central pathological mechanism underlying various cardiovascular diseases. Revolutionary advances in single-cell multi-omics technologies, such as single-cell transcriptomics, single-cell epigenomics, and spatial transcriptomics, have overcome the limitations of traditional bulk omics approaches. These advances now allow systematic dissection of metabolic heterogeneity, dynamic changes, and intercellular communication in cardiac cells at single-cell resolution under both physiological and pathological conditions. This review summarizes recent progress in the field, revealing how metabolic reprogramming drives the progression of conditions such as heart failure and myocardial infarction. It also discusses emerging metabolically targeted intervention strategies, including modulation of lactate shuttle, lipotoxicity, and immunometabolism. Finally, we critically assess the challenges in translating these findings into clinical practice and outline future directions, emphasizing the importance of interdisciplinary collaboration to advance precision medicine in cardiology.

## 1 Introduction

Cardiac metabolic reprogramming represents a central pathophysiological mechanism in various cardiovascular conditions, including heart failure, ischemia-reperfusion injury, and diabetic cardiomyopathy (Sun Q. et al., 2024). In the healthy adult myocardium, energy metabolism is highly adaptable, primarily relying on fatty acid oxidation for ATP production. Under disease conditions, however, cardiac stromal cells undergo significant metabolic shifts—such as impaired fatty acid oxidation, enhanced glycolysis, and increased ketone body utilization—which collectively contribute to an energy deficit, accumulation of lipotoxic intermediates, and progressive functional decline (Lopaschuk et al., 2021). Recent studies further indicate that metabolic reprogramming extends beyond energy supply–demand imbalance, actively participating in cell fate decisions, immune-inflammatory regulation, and tissue remodeling, thereby forming an intricate pathological network (Andreadou et al., 2025).

Although traditional bulk omics approaches have advanced our understanding of metabolic pathways, they are unable to resolve the highly heterogeneous cellular populations and their dynamic interactions within cardiac tissue. This limitation has hindered a cell-type-specific understanding of metabolic reprogramming (Perou et al., 2000; Guinney et al., 2015; Neubauer, 2007). The rapid development of single-cell multi-omics technologies—such as single-cell transcriptomics (scRNA-seq), single-cell epigenomics (scATAC-seq), single-cell metabolomics (scMetabolomics), and spatial transcriptomics (e.g., Stereo-seq)—now enables systematic characterization of cardiac metabolic remodeling at single-cell resolution, across space and time, and within molecular regulatory networks. scRNA-seq identifies cellular subpopulations and state transitions (Tian et al., 2025), scATAC-seq reveals transcriptional regulation of metabolic genes (Dunkenberger et al., 2025), and scMetabolomics directly captures metabolite dynamics (Malecki et al., 2025). Spatial transcriptomics (e.g., Stereo-seq) preserves tissue architecture and enables the mapping of intercellular communication networks. Integrating these multi-omics datasets using computational approaches such as WGCNA further supports the reconstruction of “gene–metabolite–phenotype” regulatory axes, offering a comprehensive understanding of cardiac metabolic reprogramming (Xing and Lin, 2025). These technologies not only deepen our insights into cardiac development, homeostasis, and disease mechanisms but also provide unprecedented opportunities for identifying novel therapeutic targets and developing precise metabolic interventions.

This review systematically summarizes recent advances in single-cell multi-omics approaches for studying cardiac metabolic reprogramming. We explore their potential to dissect disease mechanisms and facilitate therapeutic translation across multiple dimensions—including metabolic heterogeneity, intercellular metabolic crosstalk, immunometabolic interactions, and epigenetic regulation. We also address current technical challenges, such as the efficient capture of cardiomyocytes, algorithms for multi-omics data integration, and barriers to clinical translation. Future directions, such as dynamic metabolic tracing, artificial intelligence–driven multi-omics analysis, and the use of organoid and organ-on-a-chip models, are also discussed. By synthesizing multidimensional evidence, this review aims to offer new perspectives for the precise diagnosis and treatment of cardiovascular metabolic diseases, with the ultimate goal of improving clinical outcomes for patients.

## 2 Biological basis of cardiac metabolic reprogramming

### 2.1 Characteristics of cardiac energy metabolism

The biological foundation of cardiac metabolic reprogramming lies in the high flexibility and adaptability of cardiac energy metabolism. The healthy adult heart produces approximately 6 kg of ATP daily, primarily through mitochondrial oxidative phosphorylation, with fatty acid β-oxidation (FAO) accounting for 60%–90% of ATP production, and the remainder derived from oxidation of glucose and lactate (Neubauer, 2007). The healthy heart can rapidly switch energy substrates in response to nutrient availability, hormonal signals, and energy demands. Principal metabolic modes include fatty acid, glucose, and ketone body metabolism, amino acid metabolism.

Fatty acids enter cells via specific transporters such as CD36 (Glatz et al., 2024). Under the regulation of carnitine palmitoyltransferase 1 (CPT1), they enter mitochondria for β-oxidation, generating acetyl-CoA. Acetyl-CoA subsequently enters the tricarboxylic acid (TCA) cycle, producing reducing equivalents NADH and FADH_2_, which drive ATP production via oxidative phosphorylation in the electron transport chain (ETC.) (Bae et al., 2021). Another key regulatory site for fatty acid oxidation is carnitine palmitoyltransferase 1 (CPT1), located on the outer mitochondrial membrane, whose activity is inhibited by malonyl-CoA. Malonyl-CoA is synthesized in the cytosol by acetyl-CoA carboxylase (ACC) and can also be degraded back to acetyl-CoA by malonyl-CoA decarboxylase (MCD). When cellular ATP demand is low, acetyl groups are transferred from the mitochondria to the cytosol, promoting malonyl-CoA synthesis by ACC. Elevated malonyl-CoA then inhibits CPT1, thereby limiting fatty acid oxidation. Conversely, when energy demand increases, AMP-activated protein kinase (AMPK) phosphorylates and inhibits ACC, or MCD activity rises. Both mechanisms reduce malonyl-CoA levels, relieving CPT1 inhibition and promoting fatty acid oxidation (Vega et al., 2015; She et al., 2025). Glucose is taken up via glucose transporters (GLUT1/4) and metabolized through glycolysis to pyruvate. The majority enters mitochondria via the mitochondrial pyruvate carrier (MPC) and is oxidized to acetyl-CoA by pyruvate dehydrogenase (PDH), while a minor fraction is converted to lactate (Lopaschuk et al., 2021; Abel, 2004). Lactate can also be taken up via monocarboxylate transporters (MCT4) and oxidized for energy production. Following its uptake via MCT4, lactate is converted to pyruvate in cardiomyocytes by the enzyme lactate dehydrogenase (LDH). The subsequent metabolic fate of this pyruvate is essentially identical to that of pyruvate derived from glycolysis. When pyruvate originates from glucose, the process is termed “glucose oxidation”; when it is derived from lactate, it is referred to as “lactate oxidation” (Brooks, 2018). Recent research indicates that lactate likely represents a significant source of pyruvate in cardiomyocytes, in addition to glycolysis. Furthermore, lactate may possess signaling functions and, under certain conditions, can directly contribute carbon to the tricarboxylic acid (TCA) cycle independently of its conversion to pyruvate (Hui et al., 2017). Ketone bodies (e.g., βOHB) become important energy sources during fasting. Liver-derived ketone bodies include β-hydroxybutyrate (βOHB), acetoacetate, and acetone, with βOHB being the primary form utilized by cardiomyocytes. Ketone bodies enter cells via MCT1, are transported into mitochondria, and oxidized by β-hydroxybutyrate dehydrogenase (BDH1) to acetoacetate, ultimately yielding acetyl-CoA via thiolysis for entry into the TCA cycle (Cotter et al., 2013; Karwi et al., 2020). Furthermore, beyond their role as building blocks for proteins, amino acids contribute significantly to cardiac energy regulation through oxidative metabolism. Although the direct oxidation of branched-chain amino acids (BCAAs) contributes minimally to overall ATP production, their metabolites, such as branched-chain keto acids, function as important signaling molecules. These molecules can modulate the oxidation of both glucose and fatty acids, and influence the mTOR signaling pathway as well as insulin sensitivity (Li et al., 2017). This metabolic process is regulated by two key enzymes: mitochondrial branched-chain aminotransferase (BCATm) and branched-chain α-keto acid dehydrogenase (BCKDH). BCATm first transaminates BCAAs to generate branched-chain α-keto acids (BCKAs), which are subsequently oxidatively decarboxylated by BCKDH. Similar to the regulation of pyruvate dehydrogenase (PDH), BCKDH activity is inhibited by its specific kinase, BDK, and can be activated through dephosphorylation by the phosphatase PP2Cm (Sweatt et al., 2004).

**FIGURE 1:**
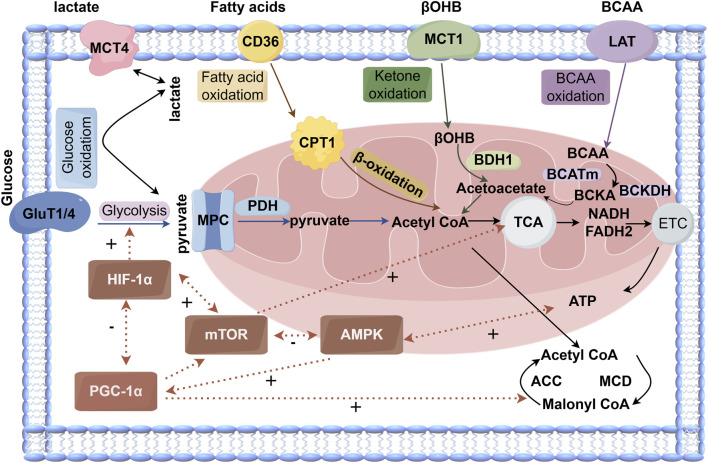
The cardiac metabolism of glucose, fatty acids, and ketones involves several major metabolic pathways. Glucose metabolism (blue) can be categorized into glycolysis and downstream pyruvate metabolism/glucose oxidation. Fatty acid oxidation (yellow) is regulated by malonyl-CoA, an endogenous inhibitor. Ketone oxidation (green) is primarily described using β-hydroxybutyrate (βOHB), the main circulating ketone body. The TCA cycle incorporates acetyl-CoA derived from these pathways and generates reducing equivalents —NADH and FADH_2_ — which enter the electron transport chain (ETC.) to drive ATP production.

Cardiac metabolic reprogramming is regulated by signaling pathways such as AMPK/PGC-1α, HIF-1α, and mTOR. AMPK senses low energy status and promotes ATP production, enhances peroxisome proliferator-activated receptor gamma coactivator 1-alpha (PGC-1α) activity and expression, and stimulates mitochondrial biogenesis and antioxidant defense (Rakoubian et al., 2025). PGC-1α serves as a master regulator of mitochondrial biogenesis (Qian et al., 2024). HIF-1α promotes glycolysis and suppresses oxidative metabolism under hypoxia (Wang et al., 2018). mTOR integrates nutrient and growth signals to regulate mitochondrial autophagy and biogenesis (Sciarretta et al., 2022). These key metabolic pathways do not operate in isolation but form a highly interconnected regulatory network. In various disease contexts, AMPK and mTOR engage in mutual inhibition: AMPK phosphorylates and inhibits mTOR to activate autophagy, while mTOR can suppress autophagy by phosphorylating the autophagy initiating kinase ULK1. This AMPK–mTOR crosstalk is crucial for regulating autophagy under energy stress and glucose deprivation (Ahn and Kim, 2013; Yang M. et al., 2025). HIF-1α and mTOR engage in positive feedback, with mTOR enhancing HIF-1α expression and HIF-1α-induced glycolysis producing ATP that further activates mTOR (Land and Tee, 2007). PGC-1α and HIF-1α antagonize each other, with PGC-1α promoting mitochondrial oxidation and HIF-1α favoring glycolysis (Viganò et al., 2011). The interplay among these pathways forms a sophisticated regulatory network whose dysregulation leads to metabolic imbalance and cardiac dysfunction (Figure 1).

Key regulatory interactions include mutual inhibition between AMPK and mTOR, positive feedback between HIF-1α and mTOR, and antagonism between PGC-1α and HIF-1α. Malonyl-CoA levels are bidirectionally controlled by ACC (positive regulation) and MCD (negative regulation). Mitochondrial pyruvate flux is gated by the mitochondrial pyruvate carrier (MPC) system. Abbreviations: GLUT1/4, glucose transporter 1/4; MCT4, monocarboxylate transporter 4; MPC, mitochondrial pyruvate carrier; ACC, acetyl-CoA carboxylase; MCD, malonyl-CoA decarboxylase; CD36, fatty acid translocase; CPT1, carnitine palmitoyltransferase 1; βOHB, β-hydroxybutyrate; BDH1, β-hydroxybutyrate dehydrogenase 1; LAT, L-type amino acid transporter; BCAA, branched-chain amino acids; BCATm, mitochondrial branched-chain amino acid transaminase; BCKDH, branched-chain α-keto acid dehydrogenase; TCA, tricarboxylic acid; NADH, nicotinamide adenine dinucleotide; FADH_2_, flavin adenine dinucleotide; ETC., electron transport chain; ATP, adenosine triphosphate; mTOR, mechanistic target of rapamycin; AMPK, 5′AMP-activated protein kinase. “+” indicates positive regulation, “–” indicates negative regulation).

### 2.2 Metabolic dysregulation in disease states

Cardiac metabolic reprogramming in cardiovascular disease has become a major focus of research in recent years. Under pathological conditions, the finely tuned balance of metabolic pathways in the adult myocardium is disrupted, leading to systemic metabolic dysregulation.

#### 2.2.1 Metabolic reprogramming in heart failure

Heart failure (HF), a complex clinical syndrome, is characterized by progressive metabolic remodeling. A shift in substrate utilization by cardiomyocytes has emerged as a central mechanism in disease progression, marked by significant alterations in substrate preference and reduced efficiency of energy metabolism (Bertero and Maack, 2018). This metabolic remodeling manifests differently in heart failure with reduced ejection fraction (HFrEF) and heart failure with preserved ejection fraction (HFpEF). HFrEF is typically associated with suppressed fatty acid oxidation and enhanced glycolysis. In contrast, HFpEF, particularly in patients with obesity and metabolic syndrome, often demonstrates excessive fatty acid oxidation activation and consequent lipotoxicity. In the early stages of HFrEF, the heart attempts to maintain energy supply through adaptive metabolic changes. Partial inhibition of fatty acid oxidation and a compensatory increase in glucose utilization help reduce oxygen consumption and preserve ATP production (Hage et al., 2020). However, as the disease advances, these adaptations become maladaptive. Uncoupling between glycolysis and glucose oxidation leads to proton accumulation and intracellular acidosis. A sustained decline in mitochondrial oxidative capacity causes an elevated NADH/NAD + ratio, which further inhibits pyruvate dehydrogenase (PDH) activity. Concurrently, the accumulation of fatty acid intermediates, such as ceramides and diacylglycerols, triggers lipotoxicity and insulin resistance (Aksentijevic et al., 2025; Liu et al., 2014; Bornstein et al., 2024). Collectively, these changes result in insufficient ATP production and impaired contractile function.

The metabolic profile of HFpEF is more complex. Recent evidence indicates an interplay between systemic metabolic inflammation and myocardial energy metabolism. In obesity-related HFpEF, adipose tissue releases excessive fatty acids and proinflammatory cytokines, including TNF-α and IL-6, leading to fatty acid overload in cardiomyocytes and mitochondrial dysfunction (Bahrami et al., 2024). The abnormal accumulation of metabolic intermediates plays a critical role in driving disease progression (Gibb et al., 2025; Luo et al., 2025).

In the failing heart, metabolites such as acetyl-CoA, succinyl-CoA, and S-adenosylmethionine influence cellular function through posttranslational modifications, including acetylation, succinylation, and methylation (Maack, 2025). For instance, hyperacetylation of mitochondrial proteins suppresses the activity of fatty acid oxidation enzymes. O-GlcNAcylation of transcription factors and ion channel proteins alters gene expression and calcium handling. Additionally, changes in histone methylation patterns reactivate fetal gene programs. These findings illustrate how metabolic disturbances directly contribute to cardiac remodeling via epigenetic mechanisms (Lehmann et al., 2018; Kronlage et al., 2019).

#### 2.2.2 Metabolic reprogramming in ischemic heart disease

Ischemic heart disease, a cardiac condition resulting from reduced coronary blood flow and inadequate oxygen supply to the myocardium, is characterized by a metabolic shift from oxidative phosphorylation toward glycolysis (Zeng et al., 2024).

During the initial phase of ischemia/reperfusion, activation of hypoxia inducible factor 1 alpha (HIF-1α) promotes glycolysis. This adaptive response ensures the myocardium maintains essential ATP production under hypoxic conditions, albeit at a significantly lower efficiency compared to complete glucose oxidation (Hu et al., 2018). With prolonged ischemia, the activity of carnitine palmitoyltransferase 1 (CPT1) decreases in the ischemic myocardium. This reduction impairs the transport of long-chain fatty acids into the mitochondria, leading to suppressed fatty acid oxidation (Gao et al., 2023). Consequently, non-oxidized fatty acids accumulate within cardiomyocytes and are converted into lipid-derived toxic substances, which subsequently induce mitochondrial damage and cardiomyocyte apoptosis (Hu et al., 2025; Li et al., 2020).

#### 2.2.3 Metabolic alterations in diabetic cardiomyopathy

Diabetic cardiomyopathy is characterized by impaired glucose utilization and excessive reliance on fatty acids, which promotes the formation of advanced glycation end products (AGEs) and a marked increase in reactive oxygen species (ROS) (Guo et al., 2025). Furthermore, recent research has identified key roles for the pyruvate dehydrogenase kinase 1 (PDK1)/Akt-GSK3β-mPTP pathway and the AARS2-PKM2 metabolic axis in regulating this metabolic shift during myocardial ischemia (Amina et al., 2025; Zhang Z. et al., 2025). High-density lipoprotein (HDL) attenuates ischemia-reperfusion injury in a scavenger receptor class B type 1-dependent manner, an effect mediated in part by its component sphingosine-1-phosphate (S1P), which has been shown to reduce infarct size (Al-Jarallah and Babiker, 2024).

Single-cell technologies now enable the detailed analysis of cell-type-specific metabolic responses in these complex settings. For instance, studies have revealed that cardiac fibroblasts are particularly sensitive to hyperglycemia, whereas cardiomyocytes are more vulnerable to damage from fatty acid overload (Zhou et al., 2024). It is important to note that metabolic reprogramming extends beyond energy production pathways, involving profound changes in anabolic and signaling metabolism. Under pressure overload, cardiomyocytes adapt the catabolism of specific amino acids, including branched-chain amino acids (BCAAs), to generate precursors for protein synthesis and antioxidant defense (Xiao et al., 2025). Additionally, glycolytic enzyme routing directs intermediates such as 3-phosphoglycerate (3-PG) into the hexosamine biosynthesis and serine-glycine pathways, thereby supporting cell proliferation and extracellular matrix production (Kashihara et al., 2022). These findings underscore the multifaceted nature of metabolic reprogramming, which addresses not only the energy crisis but also attempts to meet the biosynthetic demands of cardiac remodeling.

## 3 Methodological advances in key single-cell multi-omics technologies

Single-cell multi-omics sequencing technologies integrate data analyses across multiple molecular layers. Single-cell multi-omics sequencing technologies such as single-cell transcriptomics (scRNA-seq), single-cell epigenomics (scATAC-seq), single-cell proteomics, and single-cell metabolomics are commonly used in recent years. By capturing information from several biological levels simultaneously, these technologies overcome the limitations of bulk analysis, which often masks cellular heterogeneity. They enable the discovery of novel cell types and transitional cell states based on the unique transcriptional and epigenetic profiles of individual cells. In this section, we will systematically review key methodological advances in single-cell multi-omics technologies. Our focus will be on breakthroughs in their technical principles, the evolution from bulk to single-cell analysis, and how these advances are enhancing our understanding of the mechanisms underlying cardiac metabolic diseases. We will also briefly discuss selected studies in the cardiovascular system that have successfully integrated different single-cell multi-omics approaches.

### 3.1 Technological evolution from bulk to single-cell RNA sequencing

Since its initial demonstration in 2009, single-cell RNA sequencing (scRNA-seq) has evolved from low-throughput plate-based platforms to high-throughput droplet-based systems (Tang et al., 2009). Early scRNA-seq methods relied on laborious manual procedures and low-throughput platforms, which significantly limited their broad application. A critical advancement came in 2011 with the method developed by Islam et al., which utilized single-molecule fluorescent *in situ* sequencing (FISSEQ). This approach markedly improved detection sensitivity and quantitative accuracy, laying the groundwork for high-throughput single-cell analysis (Islam et al., 2011). A major turning point arrived in 2015 with the introduction of Drop-seq and the subsequently commercialized 10x Genomics Chromium system. These platforms employed microfluidics to co-encapsulate individual cells with uniquely barcoded beads within oil droplets. This strategy enabled efficient labeling and library construction for tens of thousands to millions of single cells, providing the technological foundation for large-scale scientific initiatives like the Human Cell Atlas (Klein Allon et al., 2015). In recent years, single-cell transcriptomic technologies have continued to advance towards higher throughput and lower cost. For instance, the Seq-Well method, developed by the Gierahn team in 2017, is a portable and low-cost platform for single-cell library preparation that further reduced the technical barriers to adoption (Gierahn et al., 2017). More recently, the newly developed UDA-seq technology increased the cell throughput per channel to over 100,000 cells—a 10- to 20-fold improvement over existing methods—while also supporting multimodal analysis, substantially reducing the cost of population cohort studies (Li Y. et al., 2025). In the field of cardiac research, optimized tissue digestion and fluorescence-activated cell sorting protocols, combined with dual fluorescent staining to ensure cell viability, have enabled high-quality transcriptomic analysis of cardiomyocytes from patients with hypertrophic cardiomyopathy. This technical progress establishes a foundation for directly investigating metabolic reprogramming in human diseased cardiac tissue (Wehrens et al., 2022).

### 3.2 Evolution of epigenomic analysis from bulk to single-cell resolution

In parallel with transcriptomic profiling, epigenomic analysis has also undergone a transition from bulk to single-cell resolution. Epigenetic regulation, particularly the study of chromatin accessibility, is critical for understanding cell identity determination and fate transitions. Prior to the advent of single-cell technologies, epigenetic studies primarily relied on bulk cell analyses using techniques such as ATAC-seq and ChIP-seq. While these bulk sequencing methods could identify key features of cis-regulatory elements, they were unable to reveal heterogeneity and dynamic changes within cell populations (Ma and Zhang, 2020).

Single-cell ATAC-seq (scATAC-seq) overcomes this limitation by optimizing the reaction conditions for Tn5 transposase within individual nuclei and combining this with microfluidic technology. This enables parallel sequencing of chromatin accessibility across thousands of cells (Lareau et al., 2021). In 2018, (Cusanovich et al. 2018) achieved the first genome-wide mapping of chromatin accessibility landscapes at single-cell resolution, laying the foundation for uncovering cell type-specific epigenetic regulation (Cusanovich et al., 2018). To directly correlate gene expression with epigenetic state within the same cell, researchers have developed multi-omics technologies such as SNARE-seq. This method uses bifunctional barcoded beads to simultaneously capture mRNA and chromatin fragments. By leveraging a shared cellular barcode, both types of information can be linked back to the same cell. This approach effectively mitigates the challenge of information loss due to signal sparsity common in single-cell multi-omics data, significantly enhancing the ability to decipher regulatory mechanisms in low-abundance cell types (Chen et al., 2019).

Collectively, these technological advances have substantially advanced our understanding of epigenetic heterogeneity and cellular relationships, providing an unprecedented perspective for dissecting aberrations in gene regulatory networks underlying cardiac metabolic diseases.

### 3.3 Single-cell proteomic and metabolomic analysis technologies

Single-cell proteomics and metabolomics represent two major branches of modern omics research, enabling the direct capture of metabolite dynamics and providing insights into cellular metabolic characteristics and alterations under pathological conditions.

In the field of proteomics, the advent of mass cytometry (CyTOF) marked a fundamental breakthrough. Conventional flow cytometry is limited by spectral overlap among fluorophores, typically allowing simultaneous analysis of only about a dozen protein markers (Bendall et al., 2011). CyTOF addresses this by using stable heavy metal isotope-labeled antibodies and combines principles of inductively coupled plasma mass spectrometry (ICP-MS) with flow cytometry. This approach virtually eliminates signal interference and enables the simultaneous analysis of over 40 proteins in a single assay (Atkuri et al., 2015). This capability allows in-depth profiling of immune cell functional states in complex tissues such as atherosclerotic plaques. For example, researchers using CyTOF to analyze multiple protein markers simultaneously observed the expansion of CD4^+^ effector memory T cells and their pro-fibrotic role in dilated cardiomyopathy. By integrating multi-omics frameworks, such as WGCNA and machine learning, diagnostic models were constructed, revealing interactions between lactate metabolism genes and T cell subsets in atrial fibrillation (Zhang X. et al., 2025).

Building on this principle, Imaging Mass Cytometry (IMC) extends these advantages to tissue *in situ*. IMC uses metal-tagged antibodies on tissue sections, which are then ablated by a laser in a raster pattern. The released metal signals are detected by CyTOF, allowing multiplexed protein imaging of dozens of targets at subcellular resolution while preserving spatial information (Glasson et al., 2023). While widely applied in tumor microenvironment studies, IMC is equally suitable for mapping the spatial distribution and interactions of different cell types in heart tissue—for instance, revealing the spatial interplay among cardiomyocytes, fibroblasts, and immune cells after myocardial infarction (Yao et al., 2023).

Beyond antibody-based detection, Olink Proteomics offers an alternative ultrasensitive solution for protein biomarker discovery. Its core technology is the Proximity Extension Assay (PEA). For each target protein, Olink designs a pair of specific antibodies, each conjugated to a unique single-stranded DNA oligonucleotide. When both antibodies bind to the target protein in close proximity, the DNA strands hybridize and are extended to form a unique DNA barcode. This barcode is then amplified and quantified via qPCR or next-generation sequencing (Babačić et al., 2020). The PEA technology achieves high-precision quantification of up to thousands of proteins using only microliter volumes of sample (e.g., 1 µL of plasma), making it particularly suitable for biomarker discovery in precious clinical samples such as endocardial biopsy fluid (Sanders-van Wijk et al., 2020).

In metabolomics, mass spectrometry-based methods are widely adopted due to their high sensitivity. The integration of stable isotope labeling strategies with single-cell metabolomic analysis represents an innovative platform that has shifted the focus from static concentration measurements to dynamic metabolic flux analysis. For instance, a dynamic single-cell metabolomics platform developed by one research team combines non-targeted stable isotope tracing with nano-flow infusion mass spectrometry. This allows global activity analysis of metabolic networks at single-cell resolution, revealing heterogeneity in pathway activities that cannot be captured by traditional concentration-based measurements (Qin et al., 2024).

### 3.4 The drive of multi-omics integration and computational methods

With the advancement of single-cell technologies, the research focus has shifted from single-omics profiling to multi-omics integration. This approach aims to capture multiple types of molecular information from the same cell, enabling a more comprehensive understanding of the regulatory mechanisms governing cell states. For instance, integrating single-cell RNA sequencing (scRNA-seq) with multiplexed error-robust fluorescence *in situ* hybridization (MERFISH) has enabled the construction of a high-resolution spatial atlas of human heart development, precisely delineating the spatial organization of cell types (Farah et al., 2024). Li et al. (2017) systematically mapped the spatial transcriptomic identities of cells in the embryonic mouse heart and developed the ATLAS-seq tool, which can trace the anatomical origin of individual cardiomyocytes with over 91% accuracy, establishing a methodological foundation for resolving cardiac cellular heterogeneity at single-cell resolution (Li et al., 2016). By integrating scRNA-seq with spatial transcriptomic data from mouse hearts after myocardial infarction, (He S. et al., 2024) uncovered the central role of lymphatic endothelial cells (LECs) as “immunometabolic regulators” in cardiac repair (He J. et al., 2024). Furthermore, integrated spatial transcriptomics (Stereo-seq) and scRNA-seq have been employed to map the cellular dynamics during zebrafish heart regeneration (Li L. et al., 2025). In a diabetic cardiomyopathy model, the combination of ^13^C -labeled glucose tracing and scRNA-seq revealed a “metabolic symbiosis” wherein enhanced lipid metabolism in cardiomyocytes coexists with increased lactate secretion from fibroblasts, collectively driving myocardial fibrosis (Zhou et al., 2024).

**FIGURE 2:**
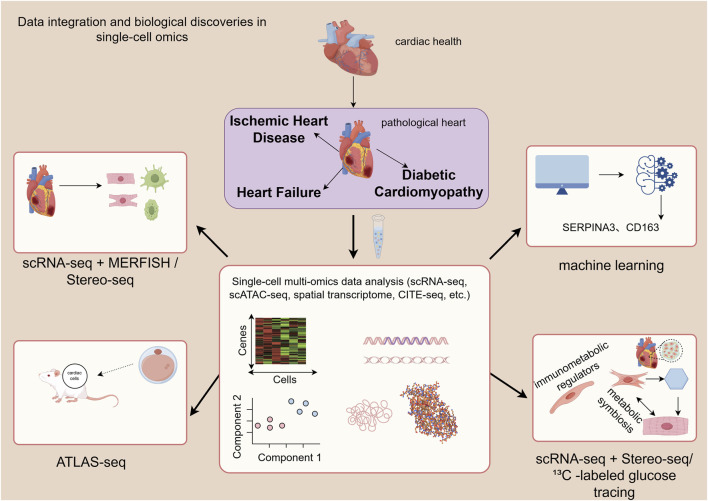
This figure illustrates the analytical pathway from multi-omics data generation to integration, demonstrating its application in revealing the spatial atlas of heart development, determining cellular origins, identifying metabolic symbiosis, and discovering biomarkers. MERFISH, multiplexed error-robust fluorescence *in situ* hybridization).

In the realm of computational methods, machine learning and computational biology are now deeply embedded in data analysis. They are used for automated cell annotation, identification of rare cell subpopulations, and the construction of diagnostic models. For example, they have been applied to screen for macrophage-associated biomarkers (such as SERPINA3 and CD163) in heart failure (Li S. et al., 2023). Notably, the recently developed computational method, single-cell Flux Estimation Analysis (scFEA), provides a flexible and powerful framework for inferring metabolic states directly from scRNA-seq data. This tool holds significant potential for dissecting cell-type-specific metabolic reprogramming in conditions like heart failure and ischemia-reperfusion injury, identifying key metabolic enzymes, and discovering novel therapeutic targets (Alghamdi et al., 2021) (Figure 2).

However, single-cell analysis of cardiac tissue still faces technical limitations. Adult cardiomyocytes are large and tightly connected, leading to low capture efficiency and transcriptional bias during dissociation (Litviňuková et al., 2020). Single-nucleus RNA sequencing (snRNA-seq) partially alleviates this issue, but nuclear transcriptomes do not fully represent cytoplasmic states (Khan et al., 2024). Tissue dissociation also results in loss of spatial information and distortion of cellular proportions (Yue et al., 2020). Multimodal data integration poses challenges due to technical variations and algorithmic limitations; computational integration methods often lack cardiac-specific optimization (Zuo and Chen, 2021; Li et al., 2022). Moreover, current approaches are costly and time-consuming, hindering clinical translation (Feng et al., 2020). Future improvements should focus on optimizing cardiomyocyte isolation methods, combining nuclear sequencing with computational deconvolution to distinguish cellular composition and state changes (Wang et al., 2020; Gural et al., 2025). Developing heart-specific analytical tools and deep learning models will advance multi-omics integration and spatial multimodal technologies, enabling *in situ* dynamic observation of cellular function (Stoeckius et al., 2018). Strengthening closed-loop computational–experimental validation, coupled with organoid and organ-on-a-chip models, will promote personalized modeling and drug screening, ultimately advancing precision diagnosis and treatment of cardiovascular diseases. Although cardiac single-cell analysis currently faces multiple technical limitations, rapid technological progress and collaborative efforts are gradually addressing these challenges. As methodologies continue to improve, single-cell analysis of the heart is expected to realize its full potential in the coming years, profoundly transforming our understanding of cardiac biology and the treatment of cardiovascular diseases.

## 4 Cardiac metabolic heterogeneity from a single-cell perspective

Single-cell analysis of the heart is a cornerstone of modern cardiovascular research, focused on decoding the molecular signatures and functional states of diverse cell types and subpopulations within this complex organ. The heart is highly heterogeneous and composed of multiple cell types, including cardiomyocytes, cardiac fibroblasts, endothelial cells, smooth muscle cells, and immune cells (such as macrophages and T cells) (Frangogiannis, 2012). Traditional bulk sequencing approaches treat cardiac tissue as a whole, providing only averaged gene expression signals and masking cellular heterogeneity. In contrast, single-cell technologies reveal distinct gene expression profiles for each cell type in the heart. Each cell type exhibits unique metabolic characteristics that sustain normal cardiac function under physiological conditions, and undergo specific alterations in disease states, driving pathological progression (Khan et al., 2024). Breakthroughs in single-cell multi-omics technologies (e.g., scRNA-seq, snRNA-seq, scATAC-seq, spatial transcriptomics) have enabled unprecedented resolution in dissecting this metabolic heterogeneity, offering new insights into the mechanisms of heart disease.

### 4.1 Cell type-specific metabolic features in the healthy heart

Cardiomyocytes, the primary functional units of the heart, account for 70%–80% of its volume and have high energy demands, relying mainly on mitochondrial oxidative phosphorylation (OXPHOS) to produce ATP. Metabolic heterogeneity among cardiac cells is not static but is established and refined during heart development. For instance, embryonic cardiomyocytes primarily depend on glycolysis for energy production. As the heart matures, the metabolic program shifts toward fatty acid oxidation. This transition is tightly regulated by transcription factor networks (e.g., the PPARα/PGC-1α axis) and epigenetic mechanisms (Li et al., 2024). In healthy adult mammalian hearts, cardiomyocytes preferentially utilize fatty acids (accounting for 60%–90% of energy production), followed by glucose, lactate, and ketone bodies. This metabolic flexibility is essential for sustaining efficient contraction (Lesnefsky et al., 2016).

In contrast, cardiac fibroblasts are primarily responsible for synthesizing and maintaining the extracellular matrix (ECM). In their quiescent state, they mainly rely on OXPHOS to meet energy needs but exhibit metabolic plasticity. During collagen synthesis, they require large amounts of amino acids (e.g., proline) and nucleotide precursors (Baudino et al., 2006). Cardiac endothelial cells line the blood vessels and regulate the delivery of nutrients and oxygen. They display active glycolytic metabolism even under oxygen-rich conditions—a phenomenon known as “aerobic glycolysis” or the Warburg effect. This is thought to conserve oxygen for diffusion into surrounding tissues while providing biosynthetic precursors for angiogenesis (Xie B. et al., 2023). Cardiac immune cells, such as macrophages, exhibit metabolic states that directly determine their functional phenotype: pro-inflammatory (M1-like) macrophages rely on glycolysis for rapid ATP production and synthesis of inflammatory mediators, whereas reparative (M2-like) macrophages depend more on fatty acid oxidation (FAO) and OXPHOS to support long-term tissue repair functions (Tung et al., 2025).

### 4.2 Cell type-specific metabolic reprogramming in the cardiac disease microenvironment

The development and progression of cardiovascular diseases are closely linked to metabolic interactions within the cardiac microenvironment. These interactions are based on the distinct metabolic profiles of different cell types. Single-cell multi-omics studies have revealed that under disease conditions, various cell types undergo specific metabolic reprogramming patterns. These changes not only affect cell-autonomous functions but also reshape microenvironmental homeostasis through metabolite exchange.

**FIGURE 3:**
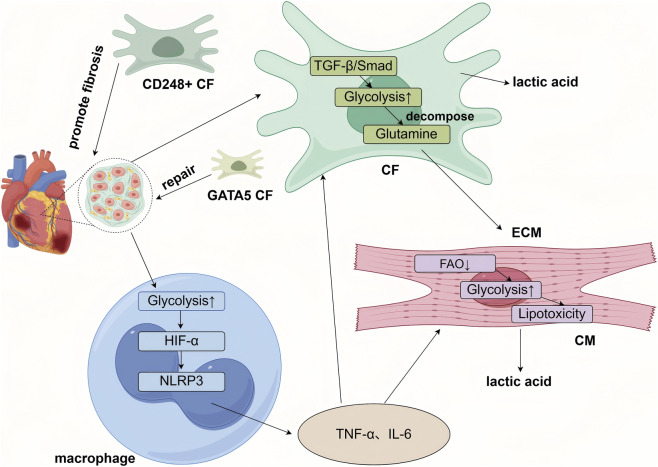
Cell-type-specific metabolic reprogramming within the cardiac microenvironment underlies the progression of heart disease. Briefly, cardiomyocytes (CMs) shift toward a fetal-like metabolic pattern characterized by enhanced glycolysis and reduced fatty acid oxidation (FAO), leading to lipotoxicity, energy deficiency, and functional decline. Cardiac fibroblasts (CFs) adopt a synthetic metabolism with increased glycolysis and glutaminolysis, which activates profibrotic pathways such as TGF-β, promotes their differentiation into myofibroblasts, and drives excessive extracellular matrix (ECM) deposition, resulting in fibrosis. Single-cell studies have revealed functionally distinct CF subpopulations, including profibrotic CD248-positive subsets and repair-prone subsets expressing GATA5 or ISL1. Macrophages exhibit heightened glycolysis and, through pathways involving HIF-1α, secrete proinflammatory cytokines such as TNF-α and IL-6. These immune-derived cytokines further suppress energy metabolism in cardiomyocytes and promote fibroblast activation. Activated fibroblasts contribute to impaired cardiac function via ECM remodeling. In turn, lactate produced by cardiomyocytes and ongoing inflammatory signals exacerbate immune cell activation, creating a vicious cycle in the diseased heart).

Pathological stressors (e.g., pressure overload, ischemia, metabolic syndrome) disrupt baseline metabolic programs, induce metabolic reprogramming, and give rise to new cell subpopulations with distinct metabolic features. These subpopulations are key drivers of disease progression. Single-cell multi-omics technologies allow precise identification of these subsets and elucidate the role of their metabolic traits in disease mechanisms (Figure 3).

#### 4.2.1 Metabolic reprogramming in cardiomyocytes

Under pathological conditions, cardiomyocytes undergo metabolic reprogramming as an adaptive response to stress (e.g., ischemia, hypoxia). Single-cell multi-omics studies show that this reprogramming is not uniform—even among cardiomyocytes, there is heterogeneity in metabolic state, function, and disease response. A subset of cardiomyocytes exhibit “metabolic dedifferentiation,” re-expressing fetal metabolic genes and reverting to a glycolytic metabolism while suppressing glucose oxidation (Zhu et al., 2021). Single-cell multi-omics studies have revealed that non-proliferating mononuclear diploid cardiomyocytes (CM4) intrinsically exhibit lower mitochondrial metabolic activity and higher glycolytic flux. This inherent metabolic profile may predispose them to dedifferentiation under pathological stress (Zhu et al., 2023). In the short term, this metabolic shift may serve as an adaptive survival strategy during energy crisis, particularly in hypoxic conditions. However, over the long term, since glycolysis produces ATP far less efficiently than oxidative phosphorylation, it ultimately leads to insufficient energy supply. Concurrent accumulation of lactate contributes to acidosis and oxidative stress, collectively accelerating the deterioration of cardiac function (Akki et al., 2008). Some studies show that inhibiting fatty acid oxidation (e.g., via CPT1B inactivation) can induce cardiomyocyte dedifferentiation, proliferation, and heart regeneration, accompanied by accumulation of α-ketoglutarate and epigenetic remodeling mediated by KDM5. This demonstrates that altering cardiomyocyte energy metabolism can reverse maturity, suggesting a potential regenerative strategy (Li X. et al., 2023).

In contrast to cardiomyocytes undergoing metabolic dedifferentiation, another subset of cardiomyocytes exhibits primarily dysregulated fatty acid metabolism in response to pathological stress. In these cells, the PPARα signaling pathway is suppressed, resulting in reduced fatty acid oxidation (FAO) capacity (She et al., 2025). Consequently, non-oxidized fatty acids accumulate within cardiomyocytes as triglycerides, creating a lipotoxic environment. These lipotoxic metabolites establish a vicious cycle: impaired fatty acid metabolism leads to the buildup of lipid-derived toxic species, which further suppress fatty acid oxidation and push cardiomyocytes toward greater reliance on glucose for energy production. However, as the disease progresses, glucose utilization eventually becomes impaired alongside mitochondrial dysfunction, culminating in a global energy crisis. This dysregulation is particularly evident in diabetic cardiomyopathy models, where myocardial fatty acid uptake is increased, yet mitochondrial fatty acid activation and oxidation efficiency are compromised (Wu T. et al., 2025). Furthermore, studies have revealed that failing cardiomyocytes shift from a mature state characterized by MYH6 expression toward a disease-associated state co-expressing NPPA/NPPB (atrial and brain natriuretic peptides) and ADGRL3. This phenotypic convergence is accompanied by broad downregulation of metabolic pathways, including oxidative phosphorylation and fatty acid metabolism. Pseudotime trajectory analysis further indicates that failing cardiomyocytes tend to progress toward two terminal differentiation states, suggesting a loss of cellular plasticity under disease pressure and confinement to a limited set of maladaptive end phenotypes. This phenomenon of “convergent remodeling” implies that reversing or intervening in these shared disease states could represent a novel therapeutic strategy for restoring cardiac function (Koenig et al., 2022).

#### 4.2.2 Metabolic reprogramming in cardiac fibroblasts

Cardiac fibroblasts are the most abundant non-myocyte population in the heart and are primarily responsible for maintaining ECM homeostasis under physiological conditions (Tallquist, 2020). In disease states (e.g., myocardial infarction, ischemic cardiomyopathy), they undergo significant metabolic reprogramming, involving fundamental changes in energy substrate utilization (Zhang J. et al., 2025). Single-cell multi-omics studies have revealed core features of this process, including imbalances in substrate usage, abnormal energy metabolism, and altered cellular function.

Under pathological conditions, cardiac fibroblasts show clear shifts in substrate preference. While healthy fibroblasts mainly rely on fatty acid β-oxidation, diseased fibroblasts enhance glycolysis and glutaminolysis, adopting a “synthetic” metabolic state. This metabolic switch activates pro-fibrotic signaling pathways (e.g., TGF-β/Smad) through changes in ATP/AMP and NAD^+^/NADH ratios, forming a vicious cycle. For example, some fibrotic hearts show impaired fatty acid use along with increased glucose uptake and enhanced branched-chain amino acid metabolism. Mitochondrial dysfunction is also prominent in pathological fibroblasts. Reduced activity of electron transport chain (ETC.) complexes I/III leads to ROS burst. Interestingly, fibroblasts maintain inefficient OXPHOS by upregulating uncoupling protein 2 (UCP2), a form of “metabolic laziness” that promotes transformation into myofibroblasts. Moreover, downregulation of the mitochondrial pyruvate carrier (MPC) prevents pyruvate from entering the TCA cycle, leading instead to lactate production (Zhang J. et al., 2025). This finding is supported by research from Professor Li Wang’s team at Wuhan University, who showed that downregulating stearoyl-CoA desaturase 1 (SCD1) enhances fatty acid oxidation and promotes cardiac reprogramming (Jia et al., 2025). Additionally, aberrant glycolytic function is observed due to upregulation of pyruvate kinase M2 (PKM2), an isoform with lower activity than PKM1. By slowing the conversion of phosphoenolpyruvate (PEP) to pyruvate, PKM2 maintains a pool of glycolytic intermediates that serve as precursors for ECM synthesis. Studies show that fibroblast-specific switching from PKM2 to PKM1 (fbPkm2→1) improves ventricular dilation, ejection fraction, and reduces lung congestion in male mice. This protective effect is linked to enhanced mitochondrial respiration (Wells et al., 2025).

Single-cell multi-omics technologies have greatly advanced our understanding of fibroblast heterogeneity. Studies show that cardiac fibroblasts are not a uniform population but consist of functionally distinct subtypes with different metabolic features and roles in disease. Using scRNA-seq and snRNA-seq, researchers have identified multiple fibroblast subpopulations in normal and diseased hearts. For example, CD248^+^ fibroblasts were identified by Professor Xinyang Hu’s team as a subpopulation specifically involved in adverse remodeling during the mid-to-late stages after myocardial infarction. These cells, expressing high levels of CD248 and Ackr3, are mainly located in the infarct and border zones (Li G. et al., 2025). They interact with T cells to sustain a pro-inflammatory microenvironment and drive fibrosis. Mechanistically, CD248 stabilizes TGFβRI and inhibits ACKR3 degradation, promoting T cell infiltration and inflammation. Another subpopulation, GATA5/ISL1^+^ fibroblasts, was identified by Professor Xiang Zhou’s team as having reparative functions. These cells are mainly found in the infarct border zone and can differentiate into cardiomyocytes (Zhang et al., 2025d). GATA5/ISL1 promotes this conversion by inhibiting Wnt/β-catenin signaling, ultimately improving cardiac structure and function in mice after myocardial infarction. Furthermore, through snRNA-seq analysis of ischemic cardiomyopathy (ICM) hearts, researchers including Qianyuan Zhang identified five fibroblast subtypes. Among these, the FB3 subpopulation was significantly expanded in ICM and had the highest fibroblast activation score. This subtype showed strong expression of ECM related genes and may be a key driver of fibrosis in ICM (Zhang et al., 2025e).

#### 4.2.3 Metabolic reprogramming in cardiac immune cells

Immune cells play a key role in tissue repair and inflammation regulation, and their functional state is highly dependent on metabolic reprogramming. In the cardiac disease microenvironment, myeloid immune cells (e.g., monocytes, macrophages) and lymphoid cells (e.g., T cells, B cells) show significant metabolic heterogeneity.

Single-cell multi-omics studies have precisely defined macrophage subpopulations in the heart by origin and function. For instance, scRNA-seq has shown that pro-inflammatory (M1-like) macrophages rely mainly on glycolysis, while anti-inflammatory (M2-like) macrophages depend on fatty acid oxidation and OXPHOS(105). This metabolic differentiation is regulated by signaling pathways such as HIF1α, AMPK, and mTOR (106). Under ischemic or metabolic stress, myeloid cells polarize toward a pro-inflammatory phenotype (CCR2^+^ macrophages) via HIF1α-dependent glycolysis, while embryonically derived TIMD4^+^ macrophages rely on OXPHOS to exert anti-inflammatory effects. The dynamic balance between these metabolically distinct subsets determines whether inflammation resolves or becomes chronic (Andreadou et al., 2025). Furthermore, studies have identified CD72-high cardiac macrophages as a pro-inflammatory subset that drives myocardial oxidative stress and apoptosis via the transcription factor Rel, a subunit of NF-κB. During the later stages after infarction, Trem2hi macrophages contribute to anti-inflammatory responses and tissue repair by clearing dysfunctional mitochondria expelled by cardiomyocytes. The functional properties of these distinct subsets—such as their propensity for glycolysis or oxidative phosphorylation—are determined by their metabolic states (Chen et al., 2023). Single-cell RNA sequencing (scRNA-seq) and single-cell ATAC-seq enable the association of metabolic heterogeneity with disease at single-cell resolution. A study on heart failure with preserved ejection fraction (HFpEF) illustrated a close relationship between metabolic reprogramming and inflammation in macrophages from a single-cell perspective. In an ApoE knockout mouse model, metabolically stressed by a high-fat diet, saturated fatty acids such as lauric acid (C12) and myristic acid (C14) activated the TLR4-NF-κB axis in cardiac macrophages. This specifically induced the release of pro-inflammatory cytokines, including TNF-α and IL-6, directly impairing cardiomyocyte relaxation. Under metabolic stress, the expansion of pro-inflammatory macrophage subsets and their secretion of inflammatory factors suppress cardiomyocyte energy metabolism, establishing a vicious cycle (Panico et al., 2024).

Single-cell multi-omics technologies have also uncovered that the functional heterogeneity of cardiac T cells and B cells is closely linked to their unique metabolic programs. Effector T cells rely on aerobic glycolysis to fuel acute inflammatory responses, whereas memory T cells and regulatory T cells (Tregs) preferentially utilize oxidative phosphorylation (OXPHOS) and fatty acid oxidation to support long-term survival and immune homeostasis. Among these, cardiac Tregs exhibit distinctive metabolic adaptability, preferentially oxidizing mitochondrial lipids and lactate. Dysregulation of this pathway is associated with heart failure (Li M. et al., 2025). The fate of B cells is also precisely regulated by metabolism. Germinal center B cell development depends on nucleotide synthesis supported by one-carbon metabolism (Wu J. et al., 2025). More critically, the lactate-pyruvate metabolic axis, mediated by the monocarboxylate transporter MCT1, directly influences the expression of AID, a key enzyme for antibody class switching, by modulating histone acetylation levels. This mechanism is abnormally active in autoimmune-related B cells, suggesting its potential role in cardiac injury (Chi et al., 2024).

Additionally, research has shown that the enzyme SARDH regulates T cell function through a “sarcine-epigenetic” axis, while the metabolite succinate acts directly as a signaling molecule. Succinate enhances mitochondrial autophagy and histone modifications, thereby promoting stem-like properties and persistence in T cells (Si et al., 2025). These findings underpin new strategies for immunomodulation via metabolic targeting. Examples include pretreating CAR-T cells with succinate to enhance their efficacy (Ma et al., 2025), or using MCT1 inhibitors to modulate the production of pathogenic antibodies by B cells (Chi et al., 2024), offering novel avenues for treating cardiovascular conditions such as myocarditis.

## 5 Metabolic interaction networks in the disease microenvironment

The “cardiac disease microenvironment” encompasses more than cardiomyocytes; it represents a complex ecosystem composed of cardiomyocytes (CMs), cardiac fibroblasts (CFs), immune cells (such as macrophages and T cells), endothelial cells (ECs), and vascular smooth muscle cells (VSMCs). Under stress conditions such as pressure overload, ischemia, and metabolic disorders, the homeostasis of this ecosystem is disrupted. Through metabolic reprogramming, each cellular component not only adapts for its own survival but also engages in extensive cross talk via metabolite exchange, signaling molecule transmission, and epigenetic regulation. These interactions collectively drive cardiac remodeling, fibrosis, inflammation, and functional deterioration (Pinto et al., 2016). Metabolic coupling is a central aspect of this cross talk, referring to the process by which different cells coordinate their metabolic states through the exchange of metabolites, energy precursors, and signaling molecules. The “Lactate Shuttle” and “Lipid Metabolite Exchange” are two of the most studied coupling mechanisms in recent research (Wang FX. et al., 2025; Wickramasinghe et al., 2022). These mechanisms not only influence cardiac energy supply but are also involved in cellular signaling, oxidative stress responses, and immune regulation. They reflect adaptive cellular responses and may also promote disease progression, playing a key role in pathological cardiac remodeling. The integration of single-cell and spatial multi-omics technologies now allows us to resolve these interaction networks at the level. This section will explore how multi-cellular metabolic interactions, specifically lactate shuttle and lipid metabolite exchange, act as core drivers of cardiovascular disease progression through cardiac metabolic reprogramming.

### 5.1 Metabolite-mediated intercellular communication

#### 5.1.1 Lactate shuttle and signaling in cardiovascular disease

Lactate has traditionally been viewed merely as the end product of glycolysis. Under pathological conditions such as cancer, inflammation, and heart failure, immune cells (e.g., T cells and macrophages) and stromal cells (e.g., cardiomyocytes) often undergo aerobic glycolysis (the Warburg effect), leading to lactate accumulation (Chen et al., 2025). However, recent studies have thoroughly revised this perspective. Lactate is now recognized as a multifunctional biological molecule that serves a triple role in the cardiovascular system: as an energy substrate, an epigenetic modulator, and a signaling molecule (Sun K. et al., 2024).

The concept of the “lactate shuttle” describes a dual role of lactate. On one hand, the accumulation of lactate as an energy substrate acidifies the microenvironment, exacerbating inflammation and fibrosis. Conversely, lactate functions as a signaling molecule and an epigenetic regulator. Through lactylation, it modifies the functions of both histone and non-histone proteins, thereby regulating gene expression and influencing processes such as immune responses, metabolic adaptation, and cellular aging (Zhang et al., 2019). Numerous studies have shown significantly elevated local lactate levels in conditions like myocardial ischemia, heart failure, and diabetes. Through “the lactate shuttle mechanism” and via protein lactylation. This metabolic reprogramming not only alters energy supply but also modulates physiological functions and pathological progression in the cardiovascular system through a complex lactate-mediated network.

As an energy substrate, lactate can enter cells through monocarboxylate transporters (MCTs, such as MCT1). Once inside the cell, it is converted to pyruvate and enters the tricarboxylic acid cycle, providing energy for cardiomyocytes and neurons. This intercellular lactate shuttle is particularly important under cardiac stress, yet it may also contribute to pathological remodeling. In myocardial hypertrophy and heart failure (HF), failing cardiomyocytes exhibit reduced fatty acid oxidation capacity and undergo metabolic reprogramming from efficient fatty acid oxidation to a fetal-like glycolytic pattern, relying instead on less efficient glucose glycolysis. This leads to lactate accumulation in the microenvironment. Furthermore, an imbalance in the lactate shuttle, in turn, exacerbates the deterioration of cardiac function (Fernandez-Caggiano and Eaton, 2021).

The discovery of histone lactylation in 2019 marked a significant expansion of lactate’s known biological functions (Zhang et al., 2019). Intracellular lactate can be converted to lactyl-coenzyme A (lactyl-CoA). Catalyzed by the acetyltransferase p300, a lactyl group is then transferred to lysine residues on histones (e.g., H3K18la), thereby altering chromatin structure and regulating gene expression. This epigenetic regulatory function plays an important role in various cardiac pathological processes (Wang N. et al., 2022). Myocardial ischemia-reperfusion injury (MIRI) is a serious complication following revascularization in patients with acute myocardial infarction, and lactate dynamics within its disease microenvironment have become a key research focus. During ischemia-reperfusion, cardiomyocytes undergo significant metabolic reprogramming, shifting from efficient fatty acid oxidation to a glycolytic-dominated energy production mode due to hypoxia. This switch leads to markedly enhanced glycolysis and substantial lactate accumulation. The accumulated lactate subsequently suppresses mitochondrial function via lactylation, exacerbating cellular damage and death (She et al., 2024). Research by Naijin Zhang et al. first identified lactylation modification at the K1897 site of the α-myosin heavy chain (α-MHC), which is crucial for maintaining the binding of α-MHC to titin and preserving cardiac contractile function (Zhang N. et al., 2023). Notably, while this study identified p300 and SIRT1 as the lactyltransferase and delactylase for α-MHC, respectively, it found that changes in their expression were not the primary cause of decreased lactylation in heart failure. The core mechanism lies in the imbalance of the lactate shuttle. During heart failure, intracellular lactate levels in cardiomyocytes decrease, reducing lactylation modification, disrupting sarcomere structure, and promoting cardiac dysfunction. This mechanism primarily stems from increased lactate efflux (via MCT4) and enhanced lactate consumption, leading to intracellular lactate deficiency. Exogenous sodium lactate supplementation or MCT4 inhibition can increase intracellular lactate levels, restore lactylation modification, and improve cardiac function. Research by the Drakos team found that mice lacking the lactate export protein monocarboxylate transporter 1 (MCT1) developed severe myocardial hypertrophy and heart failure, with lactate levels significantly elevated early in the disease. Single-cell transcriptome analysis showed upregulation of lactate metabolism-related gene clusters (e.g., LDHA, MCT4) in cardiomyocytes and downregulation of oxidative phosphorylation gene clusters (e.g., CPT1B, PPARGC1A). Increased lactate export via MCT4 exacerbated microenvironment acidification, further promoting cardiomyocyte hypertrophy and apoptosis (Cluntun et al., 2021). Furthermore, a novel MCT4 inhibitor, VB124, was shown to inhibit lactate efflux and reverse myocardial hypertrophy, offering a new direction for heart failure treatment.

Lactate also functions as a signaling molecule by activating its specific receptors, such as GPR81. For instance, in macrophages, lactate binding to GPR81 suppresses the activation of the TLR4/NLRP3 inflammasome, thereby reducing the release of pro-inflammatory cytokines like IL-1β and TNF-α and mitigating excessive inflammation (Sun et al., 2019). Another lactate receptor, GPR132, plays a significant role in the context of atherosclerosis. In atherosclerosis, high glucose promotes macrophage glycolysis and lactate production. Lactate induces macrophage senescence and foam cell formation via the GPR132-Src signaling pathway, driving plaque formation. Single-cell transcriptome analysis revealed heterogeneity in lactate metabolism among macrophage subsets. Inhibiting the GPR132-Src pathway alleviated atherosclerosis, providing a treatment strategy for diabetic cardiovascular complications independent of lipid-lowering (Ge et al., 2025).

#### 5.1.2 The role of lipid metabolite exchange in cardiac cell communication

In recent years, the mechanisms of intercellular transport and intracellular distribution during lipid metabolite exchange have become a central topic in metabolic coupling between cardiac cells. Lipid metabolite exchange coordinates energy metabolism and signaling among different cardiac cell types by transferring components such as fatty acids, phospholipids, and cholesterol, thereby maintaining cardiac homeostasis. Normal adult myocardium primarily relies on fatty acid β-oxidation for energy. However, under stress conditions like heart failure and ischemia-reperfusion, lipid metabolic reprogramming becomes an important adaptive mechanism (Lopaschuk et al., 2010). Lipids serve not only as energy substrates but also participate in cellular signaling and membrane structure construction. However, the water-insolubility of lipids necessitates their transfer between and within cells via transport systems mediated by apolipoproteins (e.g., APOE) and membrane proteins, with APOE being a core mediator of lipid exchange (Mahley et al., 2009). A recent breakthrough study provided a novel molecular perspective on this lipid exchange process. Sukalskaia et al. discovered that Tweety Homolog 2 (TTYH2) forms a specific complex with apolipoprotein E (APOE) within the endosomal lumen, directly mediating the transfer of lipids from APOE-lipoprotein particles to the cell membrane (Sukalskaia et al., 2025). Using proteomics, cryo-electron microscopy (Cryo-EM), and single-molecule force spectroscopy, the study confirmed the structural basis of the TTYH2-APOE interaction. TTYH2 binds to the C-terminus of APOE via its extracellular domain, forming a hydrophobic cavity that provides a physical pathway for lipid diffusion from lipoprotein particles to the membrane phospholipid bilayer. Functional experiments further showed that TTYH2 significantly accelerates this lipid transfer process, and this function is specific to the TTYH2 family member (not TTYH1 or TTYH3). This process has potential significance for the transfer of lipids from fibroblasts to cardiomyocytes in the heart to support membrane repair and energy supply.

Under pathological conditions, cardiac lipid metabolism undergoes significant alterations, including an imbalance between fatty acid uptake and oxidation, accumulation of lipid intermediates, and lipotoxicity leading to cardiomyocyte apoptosis and dysfunction. Single-cell lipidomics studies have found active lipid exchange between endothelial cells and macrophages within atherosclerotic plaques. Oxidized phospholipids derived from endothelial cells can be taken up by macrophage scavenger receptors, promoting foam cell formation. Conversely, macrophage-derived sphingosine-1-phosphate (S1P) can act on endothelial cell S1PR1 receptors, enhancing barrier function (Wang et al., 2023a).

Researchers led by Zhang, through integrated single-cell transcriptomic, proteomic, and metabolomic analyses, discovered the central role of CD36 palmitoylation in regulating cardiomyocyte lipid metabolic homeostasis (Zhang et al., 2025f). The study found that CD36 mRNA expression significantly increased in cardiomyocytes after myocardial infarction, but its protein level did not increase proportionally due to post-transcriptional regulation. Instead, palmitoylation modification promoted its translocation to the plasma membrane, enhancing fatty acid uptake and leading to the accumulation of lipotoxic metabolites like DAG and ceramide, exacerbating mitochondrial dysfunction and insulin resistance. Further mechanistic studies showed that inhibiting CD36 palmitoylation not only reduced its membrane localization and lipid uptake but also promoted the interaction between CD36 and the mitochondrial protein PGAM5, activating mitophagy through dephosphorylation of FUNDC1 and Drp1, thereby improving mitochondrial energy metabolism and enhancing cardiac function. This study reveals the complex network of lipid metabolite exchange and lipotoxicity in the cardiac microenvironment. It analysised at the single-cell level how CD36 palmitoylation couples abnormal lipid metabolism with mitochondrial quality control, providing a new strategy for treating myocardial infarction by targeting metabolic reprogramming.

Research by Zeping Hu’s team found that in hypertrophic cardiomyopathy (HCM), the sphingomyelin metabolic pathway is abnormally activated in cardiomyocytes, producing large amounts of sphingosine-1-phosphate (S1P) and ceramide (Wang W. et al., 2022). S1P promotes fibroblast migration and collagen secretion via G protein-coupled receptors S1PR1/3, while ceramide activates the NLRP3 inflammasome in macrophages, driving IL-1β release. Spatial metabolomics analysis revealed enrichment of long-chain polyunsaturated fatty acids (such as arachidonic acid) at the junction between cardiomyocytes and macrophages. These fatty acids are precursors of eicosanoid inflammatory mediators and participate in regulating local inflammatory responses. This research reveals the potential regulatory, diagnostic, predictive, and subtyping roles of lipid metabolite exchange in HCM and provided a theoretical basis for achieving precision treatment of HCM. Single-cell multi-omics studies indicate that lactate shuttle and lipotoxicity jointly cause mitochondrial functional damage, triggering an energy crisis, which in turn reinforces glycolysis and lipid accumulation, forming a vicious cycle. Targeting lactylation modification or improving mitochondrial function may become important future strategies for cardioprotection.

#### 5.1.3 Remote regulation of the cardiac immune microenvironment by microbial metabolites

The regulation of the cardiac immune microenvironment by gut microbiota metabolites via the circulatory system has garnered widespread attention in recent years. Research by the Sancho team, combined with single-cell transcriptomics analysis, found that the gut microbiota metabolite imidazole propionate (ImP) is significantly elevated early in atherosclerosis (Mastrangelo et al., 2025). ImP reaches the heart via the circulatory system, activating the imidazoline-1 receptor (I1R, also known as the nitracrine receptor) on macrophages, stimulating the mTOR signaling pathway, and promoting the expansion of pro-inflammatory monocytes and TH1/TH17 differentiation. Clinical data showed a positive correlation between plasma ImP levels and subclinical atherosclerosis severity, indicating ImP induces atherosclerosis independently of lipid levels. The I1R antagonist AGN192403 significantly delayed plaque progression and even achieved partial reversal in high-fat diet mice. This suggests ImP can induce lesions even without elevated blood lipids and is significantly associated with subclinical active atherosclerosis in the population, possessing dual potential as both a biomarker and therapeutic target. Establishing the close association of ImP with active atherosclerosis and the contribution of the ImP-I1R axis to disease progression opens new avenues for improving early diagnosis and personalized treatment of atherosclerosis. Furthermore, I1R inhibitors like AGN192403 have shown efficacy in multiple models, indicating that future precise management of cardiovascular diseases might be achieved by intervening in the “gut microbiota–immune” axis. Moreover, another important microbial metabolite, trimethylamine-N-oxide (TMAO), promotes cholesterol accumulation and foam cell formation primarily by inhibiting ABCA1 expression in macrophages, mainly affecting the mid-to-late stages of atherosclerosis. ImP and TMAO together constitute an “early-late dual-stage drive model,” providing new targets for the diagnosis and treatment of atherosclerosis.

The above research indicates that intervening in the “gut microbiota–immune” axis holds promise for the precise management of cardiovascular diseases. Metabolites like ImP and TMAO possess potential as both biomarkers and therapeutic targets.

## 6 Mechanistic insights: from association to causation

While traditional research has primarily established correlations between cardiac metabolic reprogramming and pathological states, the emergence of single-cell multi-omics technologies is now propelling the field from mere association toward causal mechanistic understanding. By integrating data from single-cell transcriptomics, epigenomics, proteomics, and metabolomics, we can now move beyond static descriptions of metabolic reprogramming in heart disease. This integrated approach enables us to dynamically trace the causal chains through which metabolic signals drive cardiac repair and regeneration, thereby providing a robust theoretical foundation for precise therapeutic interventions. This section will focus on how single-cell multi-omics technologies are advancing cardiac metabolism research from correlative analysis to causal demonstration.

### 6.1 Key findings

#### 6.1.1 Triggers and spatiotemporal dynamics of metabolic reprogramming

Following cardiac injury, metabolic reprogramming does not occur randomly but is initiated by specific triggers in a coordinated spatiotemporal manner. Single-cell multi-omics technologies have further revealed that this process is highly heterogeneous and dynamic, providing critical insights into its underlying mechanisms.

Conventional views hold that metabolic reprogramming is a passive response of cardiomyocytes to ischemia or pressure overload. However, single-cell studies suggest that metabolic reprogramming may be an active driver of the loss of cardiac regenerative capacity. The team of Wei Eric Wang proposed the hypothesis that “energy metabolic reprogramming initiates cardiomyocyte proliferation,” suggesting that metabolic reprogramming is a cause rather than a consequence of the loss of proliferative ability in mammalian cardiomyocytes after birth (Chen et al., 2024). This view is supported by several lines of evidence. Firstly, the rapid decline in cardiac regenerative capacity occurs within the first week after birth, coinciding temporally with the window of metabolic reprogramming. Although the temporal coincidence between metabolic reprogramming and loss of proliferation is clear, the causal relationship remains uncertain. Whether metabolic reprogramming is a key regulatory mechanism controlling cardiomyocyte proliferation and regeneration has become a critical scientific question. Addressing this question could guide future research on cardiac regeneration and hold significant implications for treating cardiovascular diseases such as myocardial infarction and heart failure. Further elucidation of the regulatory mechanisms governing cardiomyocyte proliferation and regeneration, along with the design of spatiotemporally targeted interventions editing key metabolic enzymes, may lead to more effective therapeutic strategies for cardiovascular diseases.

A study in Molecular Therapy (2025) revealed that fatty acid oxidation drives cardiac reprogramming, elucidating the molecular mechanism by which stearoyl-CoA desaturase 1 (SCD1) drives cardiac metabolic reprogramming. It was found that the transdifferentiation of cardiac fibroblasts into induced cardiomyocytes (iCMs) requires metabolic switching. Downregulation of SCD1 activates fatty acid oxidation (FAO) via the PPARβ/PGC-1α axis, providing the necessary energy and acetyl-CoA for reprogramming. Inhibiting SCD1 increased iCM conversion efficiency by threefold and promoted mitochondrial biogenesis (Jia et al., 2025). This not only reveals the important role of metabolism in direct cardiac reprogramming but also offers new avenues for advancing reprogramming strategies.

The temporal dynamics of metabolic reprogramming are also highly significant. The neonatal mouse heart undergoes a rapid and fundamental shift from glycolysis to fatty acid oxidation within the first week after birth, a transition that coincides with the loss of its regenerative capacity (Fan et al., 2025). By integrating single-cell transcriptomic and metabolomic data, researchers have captured the dynamic changes in metabolic enzyme expression during this critical time window. This includes the upregulation of key fatty acid oxidation genes, such as CPT1B and PDK4, alongside the downregulation of glycolytic genes, including HK2 and PFKFB3(Zheng et al., 2025). The metabolic transition during this developmental period provides a valuable reference for understanding the metabolic reprogramming that occurs following injury in the adult heart.

#### 6.1.2 Immune-metabolic crosstalk and causal mechanisms in cardiac repair

The metabolic crosstalk between immune cells and cardiomyocytes during cardiac repair is a critical determinant of repair outcomes. Single-cell multi-omics technologies now allow us to deconstruct the molecular basis of this interaction, thereby establishing a causal link between immune cell metabolism and the process of cardiac repair.

Under conditions of cardiac injury or metabolic stress, such as in HFpEF, metabolic stress alters the cardiac immune microenvironment, particularly the activation state of macrophages, thereby affecting cardiac function (Panico et al., 2024). Cardiac macrophages sense local metabolic changes (e.g., saturated fatty acid accumulation) and influence cardiomyocyte gene expression and function by secreting inflammatory cytokines such as TNF-α and IL-6. Single-cell multi-omics analysis uncovered a macrophage-to-cardiomyocyte regulatory axis, predicting 57 cardiomyocyte genes as targets of macrophage-secreted ligands. These genes are enriched in cardiac stress pathways (e.g., hypertrophy, fibrosis, autophagy) and WNT/β-catenin signaling. In pro-inflammatory macrophages (CCR2+ subset), succinate accumulation stabilizes HIF-1α by inhibiting prolyl hydroxylases (PHDs), enhancing glycolytic gene expression and forming a “metabolic-inflammatory positive feedback loop.” This finding provides direct molecular evidence for immune-metabolic interactions in heart disease and a theoretical basis for immunometabolic therapeutic strategies.

Recent research has identified a unique cardiomyocyte subpopulation, known as Clu^+^ cardiomyocytes, which was initially discovered in the context of neonatal heart regeneration. These cells secrete the protein Clusterin, which acts to reprogram macrophage metabolism, promoting their polarization toward a reparative M2 phenotype. These M2 macrophages subsequently upregulate the secretion of BMP2, which in turn activates pro-proliferative signaling in cardiomyocytes via the BMPR1A receptor (Fan et al., 2025). This finding reveals a positive feedback loop: cardiomyocytes influence immune cell function through metabolic regulation, which then feeds back to stimulate their own proliferation.

This provides a novel causal explanation for the mechanisms underlying cardiac regeneration.

Causally validating metabolic interventions is a critical step for establishing the mechanisms of immune-metabolic crosstalk. A team led by Professor Xiaoheng Liu developed biomimetic lipid nanoparticles loaded with miR-10a. These nanoparticles are designed to specifically target inflammatory macrophages within atherosclerotic plaques. By restoring mitochondrial metabolic function and remodeling the epigenetic state, this intervention successfully reprograms macrophages toward an anti-inflammatory phenotype (Fang et al., 2025). This precise metabolic targeting not only attenuated atherosclerosis but also directly demonstrated the causal role of macrophage metabolic reprogramming in cardiovascular disease, thereby providing proof-of-concept for treatment strategies that target immunometabolism.

#### 6.1.3 Epigenetic regulation and metabolic memory: cooperative control of cell transdifferentiation by metabolic enzymes and epigenetics

Metabolism and epigenetic regulation play key roles in the development and progression of cardiac diseases. Metabolic enzymes and metabolites influence the epigenetic landscape, while epigenetic regulators reciprocally control the expression of metabolic genes. This synergistic regulation is critical during cardiac cell transdifferentiation and underlies a “metabolic memory” of cardiac injury. The “metabolic–epigenetic axis” senses intra- and extracellular metabolic states to directly reprogram the cellular epigenome, thereby driving and stabilizing fate transitions during transdifferentiation. The rapid advancement of single-cell multi-omics technologies provides an unprecedented perspective for uncovering how the interplay between metabolism and epigenetics controls cell transdifferentiation.

Metabolic intermediates such as acetyl-CoA, S-adenosylmethionine, and α-ketoglutarate (α-KG) serve as substrates or cofactors for epigenetic modifying enzymes, directly influencing chromatin state and gene expression (Li et al., 2018). For example, pathological cardiac hypertrophy under pressure overload has long been viewed as a process governed by gene expression. However, research in a pressure-overload model revealed a significant increase in α-KG levels within cardiomyocytes. Elevated α-KG enhances the activity of TET dioxygenases, thereby promoting DNA demethylation. This mechanism directly links cellular metabolic state to epigenetic remodeling (DNA hydroxymethylation), opening new avenues for “metabolic therapy” to intervene in cardiac hypertrophy (Mericskay et al., 2025). Furthermore, recent studies have identified non-canonical functions for various metabolic enzymes, which can translocate into the nucleus and participate directly in gene regulation. For instance, the glycolytic kinase PKM2 can enter the nucleus, where it modulates histone modifications to enhance the expression of genes related to cell proliferation. During heart regeneration, PKM2 influences cardiomyocyte proliferative capacity by regulating histone modifications and chromatin accessibility (Zheng et al., 2025). Similarly, ketohexokinase-A (KHK-A) may function in cardiac stress responses by phosphorylating its substrate p62 to promote Nrf2-mediated gene expression (Xu et al., 2019; Ghos et al., 2024). These metabolic enzymes facilitate rapid and precise coupling of metabolic state with transcription, efficiently driving cell transdifferentiation.

Long non-coding RNAs (lncRNAs), acting as mediators of epigenetic regulation, play a key role in integrating metabolic signals with cell transdifferentiation through mechanisms involving epigenetic modification, metabolic reprogramming, and miRNA sponging (Wu X. et al., 2025). For example, lncRNAs such as Braveheart (Bvht), which function as master epigenetic regulators during early cardiogenesis, recruit SUZ12/PRC2 to glycolytic genes, thereby maintaining the proliferative state of cardiomyocytes. This highlights the bridging role of epigenetic regulation between metabolic reprogramming and cell fate determination. The sirtuin family, comprising metabolism-sensitive histone deacetylases, holds a central position in the metabolic–epigenetic cross-talk. SIRT3 suppresses macrophage M1 polarization by regulating the α-KG/succinate ratio, whereas SIRT5 deficiency promotes PKM2 succinylation and drives inflammatory responses. By integrating metabolic reprogramming (e.g., of glucose, lipid, and glutamine metabolism) with epigenetic regulation (DNA methylation, histone modifications, ncRNAs), sirtuins dynamically balance macrophage M1/M2 polarization, enabling rapid adaptation of cell function to metabolic status (Lin R. et al., 2025).

Transient metabolic perturbations can induce long-term effects through epigenetic mechanisms, a phenomenon termed “metabolic memory,” which is crucial for sustaining gene expression in cells after transdifferentiation. Studies have shown that the histone demethylase JMJD1A influences cell transdifferentiation by regulating H3K9 methylation during cardiac aging (Alrumaihi et al., 2025). Under low glucose conditions, acetyl-CoA synthetase 2 (ACSS2) translocates to the nucleus and locally generates acetyl-CoA to activate gene expression. This nuclear translocation of metabolic enzymes may provide a structural basis for metabolic memory (Li et al., 2018).

Based on these mechanisms, understanding the fine-tuned regulatory network of cell transdifferentiation provides a foundation for exploring therapeutic strategies that target the metabolic–epigenetic axis. For instance, histone deacetylase (HDAC) inhibitors have been shown to improve cardiac hypertrophy and function (Alrumaihi et al., 2025). Combining SIRT1 activators (e.g., resveratrol) with DNMT inhibitors is considered a promising therapeutic direction for cardiac diseases (Flowers and Duric, 2025). SIRT6 counters fibrosis through a dual mechanism: it deacetylates Smad3 to block TGF-β signaling and directly reduces H3K9ac levels at the TGF-β promoter (Cozzolino et al., 2025), highlighting the therapeutic potential of targeting this axis (Alrumaihi et al., 2025). Nanocarrier delivery systems enable precise intervention, offering technical support for targeting the metabolic–epigenetic axis. For example, lipid nanoparticles loaded with miR-10a can reprogram macrophages to an anti-inflammatory phenotype (Fang et al., 2025). Similar strategies could be adapted to regulate cardiac cell transdifferentiation.

In summary, the synergistic regulation by metabolism and epigenetics is a core mechanism governing cell transdifferentiation. Single-cell multi-omics technologies provide a powerful toolkit for deciphering this complex network. Future efforts should focus on further elucidating cell-type-specific regulatory mechanisms, developing precise targeting strategies, and advancing the clinical translation of cardiac regeneration therapies.

### 6.2 Experimental validation cases: functional studies combining gene editing (CRISPRi/a) and metabolic flux analysis

Hypotheses generated from single-cell multi-omics require validation through meticulous functional experiments. Recently, the combination of CRISPR interference/activation (CRISPRi/a) technology with metabolic flux analysis has provided powerful tools for validating the function of cardiac metabolic regulators. CRISPRa systems enhance target gene expression by recruiting transcriptional activation domains to specific gene promoters, while CRISPRi systems repress gene transcription by recruiting repression domains (Lv et al., 2022). Integrating these tools with single-cell transcriptomic and epigenomic data enables precise manipulation and functional validation of cardiac metabolic pathways. For example, researchers used CRISPRi to knock down lncRNA TINCR expression, finding reduced ACLY protein levels, decreased acetyl-CoA production, lowered histone acetylation levels, and ultimately suppressed expression of hypertrophy-related genes (Shen et al., 2023). Conversely, using CRISPRa to activate specific genetic programs in GATA5/ISL1+ fibroblasts promoted cardiac repair and improved function after myocardial infarction (Zhang et al., 2025d). Metabolic Flux Analysis (MFA) provides direct evidence for the function of these regulators. Using stable isotope-labeled metabolites (e.g., ^13^C-glucose or ^13^C-glutamine), researchers can track the flow and conversion rates of nutrients in specific cardiac cell types. For instance, after knocking down lncRNA AC020978, MFA showed reduced flux of glucose into the TCA cycle and decreased AKT signaling activity in lung cancer cells, confirming the critical role of this lncRNA in metabolic reprogramming. The introduction of machine learning algorithms further enhances the predictive power of these studies. By integrating multi-omics data and metabolic models, researchers can predict metabolic phenotypes following specific genetic perturbations and guide the design of CRISPR screening experiments. This combined computational and experimental approach significantly accelerates the functional validation of cardiac metabolic regulators. The team led by Jared M. Churko employed CRISPR/Cas9 genome editing to knock out key transcription factors, NR2F2 and HEY2, which were initially identified through single-cell RNA sequencing. Functional experiments demonstrated that loss of HEY2 resulted in downregulated expression of the ventricular marker MYL2, accompanied by a reversal of maturation in calcium handling and action potential profiles, leading to an atrial-like phenotype. In contrast, knockout of NR2F2 promoted ventricular-like characteristics. These findings not only validated the regulatory hierarchy predicted by single-cell RNA sequencing but also provided deeper mechanistic insight, elucidating the pivotal causal role of specific transcription factors in driving subtype-specific metabolic reprogramming—such as mitochondrial function—and electrophysiological maturation in cardiac cells (Churko et al., 2018).

Single-cell multi-omics technologies have elevated the study of cardiac metabolic reprogramming from descriptive correlation to causal analysis. This transition has been made possible by the integration of multiple advanced methodologies—such as CRISPR interference and activation (CRISPRi/a), metabolic flux analysis, and machine learning—which together form a robust system for precise functional validation. By deeply integrating high-resolution molecular data with innovative experimental approaches, we are now able to systematically dissect complete causal networks, spanning from metabolic triggers to immunometabolic crosstalk, epigenetic memory, and mitochondrial function. Moreover, these tools enable precise interventions at the level of specific gene programs and metabolic fluxes, revealing cell type-specific reprogramming mechanisms in the heart. This progress establishes a solid theoretical and experimental foundation for cardiac regeneration therapies based on metabolic regulation. Future research will need to focus on further resolving the spatiotemporal dynamics and cell-type specificity of metabolic reprogramming, as well as developing innovative treatment strategies that can target specific metabolic pathways or modulate metabolic–epigenetic crosstalk. With ongoing advancements in single-cell multi-omics, we anticipate the revelation of cardiac metabolic reprogramming mechanisms at even higher resolution, ultimately bringing us closer to realizing metabolism-targeted regenerative therapies for heart disease.

## 7 Therapeutic frontiers

Recent single-cell multi-omics analyses of cardiac tissue have progressively revealed distinct metabolic reprogramming features in various cellular subpopulations—including cardiomyocytes, fibroblasts, and immune cells—under metabolic disease conditions. These findings establish a molecular basis for developing precision-targeted strategies directed at specific cell subtypes. This section is organized into two parts. First, it will discuss recent advances in targeted therapeutic research related to cardiomyocytes and fibroblasts, driven by single-cell technologies. It will then explore the latest breakthroughs in treatment strategies focusing on immune cell subpopulations.

### 7.1 Precision targeting strategies

#### 7.1.1 Precision regulation of cardiomyocyte energy metabolism

As the core of cardiac energy metabolism, cardiomyocytes undergo significant metabolic adaptations under pathological conditions, such as a shift from fatty acid oxidation to glycolysis. While beneficial in the short term under hypoxic conditions, this shift leads to energy deficiency and lipotoxicity over time. Precise regulation of cardiomyocyte energy metabolism must account for disease stage, etiology, and metabolic crosstalk between cells.

Several cardiomyocyte-specific metabolic regulatory strategies have recently been proposed. Fatty acid oxidation inhibitors (e.g., trimetazidine, ranolazine) can improve myocardial energy efficiency in heart failure; however, future studies should use single-cell technologies to evaluate their subpopulation-specific effects and avoid off-target effects (Marzilli et al., 2019; Kourampi et al., 2023). Metformin improves insulin sensitivity and mitochondrial function via AMPK signaling (Maleki et al., 2025), though its cardioprotective effects in non-diabetic heart failure patients require further validation through prospective RCTs. SGLT2 inhibitors (e.g., dapagliflozin) improve myocardial energy status by promoting ketone body utilization and ion homeostasis (Panico et al., 2024). Research led by Lei Song and Zeping Hu has shown that patients with hypertrophic cardiomyopathy (HCM) exhibit not only enhanced glycolysis and suppressed fatty acid oxidation, but also alterations in cysteine-methionine, vitamin B6, and sphingolipid metabolism, suggesting that targeting the pentose phosphate pathway and oxidative stress may represent a potential therapeutic strategy (Wang W. et al., 2022).

Accumulating evidence indicates that disruptions in myocardial fuel metabolism and bioenergetics contribute to the development of various heart diseases. Therefore, identifying changes in energy metabolism after myocardial infarction (MI) and optimizing the metabolic remodeling response are crucial. This approach may provide insights into underlying pathophysiology and offer innovative treatment opportunities for MI patients. Post-MI, dysregulated energy metabolism and mitochondrial damage can lead to structural and functional cardiac abnormalities. Studies have shown that CD36 palmitoylation mediates dysregulated fatty acid transport, leading to lipid accumulation in cardiomyocytes and impaired mitophagy, exacerbating energy crisis and cell death. Inhibiting CD36 palmitoylation reduces fatty acid overload and enhances the CD36–PGAM5–Fundc1 pathway to promote mitophagy, thereby improving cardiac function—a promising new direction for MI treatment (Zhang et al., 2025f).

Furthermore, single-nucleus multi-omics studies (snRNA-seq and snATAC-seq) have revealed that inhibition of miR-200 induces the emergence of a rare cardiomyocyte subpopulation (CM4), suggesting that miR-200 influences cardiac development and cellular maturation by regulating transcription factors and metabolic pathways. This finding offers new mechanistic insights into congenital heart disease and identifies miR-200 as a potential target for cardiac regeneration or metabolic heart disease (Leonard et al., 2025).

#### 7.1.2 Metabolic interventions targeting pro-fibrotic fibroblasts

Under pathological conditions, cardiac fibroblasts transform into myofibroblasts and undergo metabolic reprogramming, such as a shift toward aerobic glycolysis. This shift not only supplies substrates for collagen synthesis but also influences the microenvironment via lactate secretion (Zeng et al., 2022). Single-cell studies show that activated fibroblasts exhibit enhanced glycolysis and glutaminolysis. High expression of glutaminase (GLS) promotes α-ketoglutarate production, activating prolyl hydroxylase and accelerating collagen cross-linking. Glutaminase inhibitors (e.g., CB-839) reduce α-ketoglutarate and NADPH levels, suppress oxidative stress and mTOR signaling, and thereby inhibit fibrosis (Zhang J. et al., 2025; Song et al., 2019; Gibb et al., 2019). However, achieving cell-specific targeting remains challenging. Future efforts should focus on ensuring that such drugs selectively act on pathological fibroblasts without affecting normal cells that depend on glutamine.

Studies also indicate that macrophages and fibroblasts form an inflammatory loop via IFN-β–IFNAR–STAT1 signaling, which inhibits GATA4 binding and impedes fibroblast reprogramming into cardiomyocytes. This process involves not only transcriptional regulation but may also suppress cell fate transitions by altering cellular metabolic states (Wang et al., 2024). Spatial transcriptomics has revealed that endothelial cells and fibroblasts drive fibrosis and metabolic remodeling via the SCARB1 receptor (Katsuki et al., 2025). Additionally, in MI models, a GATA5/ISL1^+^ fibroblast subpopulation with cardiomyocyte-like features has been identified. These cells promote the expression of cardiac genes by inhibiting the Wnt/β-catenin pathway, improving cardiac function and reducing fibrosis, thus offering a new target for regenerative therapy (Zhang et al., 2025g).

### 7.2 Metabolic immunotherapy

Cardiac metabolic diseases are characterized by a state of chronic, low-grade inflammation, in which the immune microenvironment is intricately linked to metabolic status (Sun et al., 2021). Single-cell technologies have further elucidated how metabolic reprogramming in various immune cell types influences disease progression.

#### 7.2.1 T Cell metabolic reprogramming

T cells play a dual role in cardiac inflammation and repair, and their differentiation and functional states are finely regulated by metabolic pathways. Different T cell subsets have distinct metabolic profiles: effector T cells rely on glycolysis, Treg cells prefer fatty acid oxidation, and memory T cells depend on oxidative phosphorylation (Bansal et al., 2017). Based on these observations, several T cell metabolic reprogramming strategies have been explored. PPAR-α agonists (e.g., fenofibrate) or metformin enhance Treg function (Blanton et al., 2019); mTOR inhibitors (e.g., rapamycin) or glycolysis inhibitors reduce effector T cell inflammatory activity—though their potential impact on anti-pathogen immunity must be carefully weighed in clinical applications (Wang Z. et al., 2025). PD-1 signaling influences T cell function by modulating metabolic status, a discovery that reveals a potential metabolic mechanism for the cardiotoxicity of immune checkpoint inhibitors and suggests that metabolic interventions may mitigate these effects (Kojic et al., 2025). Additionally, the local cardiac metabolic environment (e.g., high lactate, low glucose) shapes T cell metabolic characteristics and must be considered (Wang X et al., 2025).

#### 7.2.2 Macrophage metabolic regulation

Macrophages are key components of the cardiac immune microenvironment, and their polarization and function are closely linked to local metabolites (Martini et al., 2019). Several macrophage metabolic reprogramming strategies informed by single-cell studies are currently under development. PPAR-γ agonists (e.g., pioglitazone) promote M2 polarization and facilitate cardiac repair (Wang S. et al., 2023; Konegawa et al., 2022); mitochondrial function modulators (e.g., nicotinamide mononucleotide, NMN) increase NAD^+^ levels, activate SIRT1/SIRT3, suppress NF-κB, promote M2 marker expression and repair functions, reduce collagen deposition, improve mitochondrial function and myocardial energy metabolism, and inhibit oxidative stress and cell death (He S. et al., 2024; Mann et al., 2025; Fu et al., 2024); mitochondrial transplantation into macrophages promotes a shift toward the M2 phenotype by activating fatty acid oxidation and oxidative phosphorylation, enhancing migration and phagocytosis capacity, and improving cardiac function and ventricular remodeling after MI through intercellular mitochondrial transfer (Zhang Y. et al., 2025). Modulating cholesterol metabolism—e.g., with LXR agonists—promotes cholesterol efflux, reduces foam cell formation, and enhances anti-inflammatory function, a strategy already showing promise in atherosclerosis (Liu et al., 2024; Maguire et al., 2019). Targeting inflammatory cytokines secreted by cardiac macrophages (e.g., the IL-1β inhibitor canakinumab) can indirectly improve myocardial metabolism (Wu et al., 2021; Amrute et al., 2024). The CANTOS trial demonstrated that canakinumab significantly reduces cardiovascular event risk by inhibiting IL-1β and lowering hsCRP, providing clinical proof-of-concept for metabolic immunotherapy (Ridker et al., 2018). Single-cell lipidomics has revealed that sphingomyelin and cardiolipin metabolic remodeling during M2 polarization can inhibit the NLRP3 inflammasome, suggesting novel nodes for intervention.

Integrating spatial metabolic imaging with time-resolved tracing technologies, future studies should further elucidate the spatiotemporal mechanisms of mitochondria–nucleus metabolic crosstalk in cardiac repair.

#### 7.2.3 Metabolic targeting of other immune cells

Beyond T cells and macrophages, single-cell multi-omics studies have revealed that other immune cells—including neutrophils, dendritic cells, and B cells—also contribute to shaping the immunometabolic microenvironment in the heart (Simões and Riley, 2022).

In diabetic cardiomyopathy, neutrophils undergo metabolic remodeling by taking up lactate via MCT1. Glycolysis inhibition redirects metabolic flux to the pentose phosphate pathway (PPP), driving NETosis and exacerbating myocardial injury. This suggests that targeting PDK4 (e.g., with the PDK4 inhibitor pitavastatin) or MCT1 may have therapeutic value (Yang S. et al., 2025). Dendritic cell functional states depend on a balance between glycolysis and fatty acid oxidation (Zhuang et al., 2022; Sen et al., 2023). While FAO inhibitors (e.g., etomoxir) can ameliorate myocarditis, they may impair systemic immune surveillance, highlighting the need for heart-specific targeted delivery systems (Tang et al., 2025).

B cells actively shape the immune microenvironment in metabolic cardiovascular diseases via antigen presentation. Studies show that high homocysteine levels enhance MHC II expression in B cells via the PKM2–CREB1–CIITA axis, promoting T cell activation and inflammation (Ma et al., 2022). This discovery not only reframes our understanding of B cell function in metabolic heart disease but also suggests that targeting B cell immunometabolic reprogramming may be a novel strategy to modulate the cardiac immune microenvironment.

Single-cell multi-omics technologies have profoundly revealed the cellular heterogeneity and interaction networks within cardiac immunometabolism, providing a solid theoretical foundation for developing precision therapeutic strategies.

## 8 Technology-driven therapies

Technological innovation is a key driver in translating basic discoveries in cardiac metabolic reprogramming into therapeutic applications. Advances in fields such as single-cell multi-omics, nanotechnology, gene editing, and bioengineering have provided new tools and methods for the treatment of cardiac metabolism.

### 8.1 Single-cell data-guided personalized metabolic intervention

Progress in single-cell multi-omics technologies has shifted the focus from bulk analysis to individualized profiling of cardiac cells, providing a molecular basis for developing personalized metabolic intervention strategies. By integrating single-cell data with other omics data from individual patients, patient-specific metabolic network models can be constructed to guide treatment decisions and drug selection.

#### 8.1.1 Patient-specific metabolic pathway modeling

Traditional omics studies, often based on population-level averages, overlook inter-individual heterogeneity. Single-cell multi-omics technologies now enable the construction of patient-specific metabolic network models. These models facilitate the analysis of myocardial metabolic reprogramming at an individual level, help identify dysregulated pathways, and predict potential therapeutic targets. Such models integrate single-cell multi-omics data, genomic variations, and clinical phenotypes to quantify metabolic flux changes in different cell types under pathological conditions, thereby linking genotype to metabolic phenotype. Using computational approaches like scFEA and Compass, it is possible to quantify the metabolic fluxes within specific cardiac cell subpopulations during states such as heart failure or ischemia, revealing when and how metabolic shifts occur in distinct cell types (Alghamdi et al., 2021; Wag et al., 2021).

Furthermore, the integration of genomic data allows these models to account for individual genetic backgrounds. By translating patient-specific genetic variants—such as single nucleotide polymorphisms or loss-of-function mutations in genes related to fatty acid transport, glycolysis, or mitochondrial function—into constraints on reaction rates within the metabolic network, researchers can simulate how these variants perturb the network. This links genotype to metabolic phenotype and helps explain the molecular basis for differences in individual treatment responses (Kodate et al., 2025). Integrating clinical parameters significantly enhances the predictive accuracy and translational potential of these models. Using cardiac imaging parameters (e.g., ejection fraction, ventricular wall thickness) and blood biochemical markers (e.g., lactate, fatty acid levels) as model output targets, and iteratively optimizing and calibrating the model parameters, substantially improves prediction reliability. This lays a foundation for the clinical application of such models. Patient-specific metabolic modeling also provides a framework for understanding individual variability in treatment response. For example, cardiac fibroblasts from different patients exhibit significant heterogeneity in their response to metabolic stress: some show enhanced glycolysis, while others demonstrate increased glutaminolysis. This heterogeneity may be a key reason for the variable efficacy of metabolic interventions (Zhang J. et al., 2025). Based on these models, researchers can simulate the effects of drugs, dietary changes, or exercise on cardiac metabolism, predict individual treatment outcomes, and advance the realization of truly personalized medicine (Gu et al., 2022).

#### 8.1.2 Dynamic metabolic network reconstruction and intervention optimization

Cardiac metabolic reprogramming is a dynamic process that evolves with disease progression and treatment. Single-cell multi-omics time-series analyses can capture this temporal evolution, reveal metabolic trajectories and disease transition points, and guide the timing of interventions (Bouman et al., 2024; Lin YT. et al., 2025). By integrating single-cell data with real-time physiological monitoring information, dynamic network models can be constructed to identify early warning signals, determine critical intervention windows, and optimize combination therapies (Dunkenberger et al., 2025). Research has demonstrated that inhibiting fatty acid oxidation after myocardial injury can reactivate proliferation in adult cardiomyocytes and promote cardiac repair, highlighting the therapeutic potential of reversing metabolic temporal analysis dynamics (Li X. et al., 2023) Such dynamic models support virtual disease progression simulations and personalized intervention predictions, contributing to dynamic precision medicine that may maximize therapeutic efficacy while minimizing side effects.

### 8.2 Multi-omics pharmacodynamic evaluation in organoids and organ-on-chip systems

Organoid and organ-on-a-chip technologies provide powerful tools for simulating human cardiac physiology and pathology in controlled environments. When combined with multi-omics analysis, they enable preclinical assessment of drug efficacy and safety. These advanced models overcome limitations of traditional 2D cell cultures and animal models, improving predictive value for clinical translation.

#### 8.2.1 Technological innovation and multi-organ integration

Heart-on-chip systems can mimic key features of the cardiac microenvironment, such as mechanical stress, electrical stimulation, and biochemical gradients, and often incorporate sensors for real-time monitoring of functional parameters (Zhou C. et al., 2025; Nunes et al., 2025; Kim et al., 2022). Significant progress has been made in recent years in organoid and organ-on-chip technology, including the use of human pluripotent stem cell-derived cells, integration of multiple cell types (e.g., cardiomyocytes, fibroblasts, endothelial cells, immune cells), recreation of cell-cell interactions, vascularized structures, and interconnection of multi-organ chips to simulate inter-organ interactions and systemic pharmacokinetics (Monteduro et al., 2023; Huang et al., 2024). These systems are particularly suitable for studying communication between the heart and metabolic organs such as the liver and kidneys. Understanding inter-organ crosstalk is essential for developing more precise systemic disease treatments and effective therapeutic strategies (Liu K. et al., 2025). By recreating dynamic interactions and metabolic dialogue between the heart and other organs (e.g., liver, kidneys) in a highly biomimetic microenvironment, and leveraging metabolomics (e.g., LC-MS) and proteomics methods, researchers can track drug distribution and metabolic transformation, uncovering drug toxicity and off-target effects (Ni et al., 2025). These advances not only greatly enhance the accuracy of preclinical predictions but also establish a solid theoretical and practical foundation for precision medicine.

#### 8.2.2 Multi-omics integration and efficacy evaluation

By combining single-cell transcriptomics, proteomics, and metabolomics analyses, organoids and organ-on-chip systems provide dynamic, multi-dimensional information on drug response, going beyond traditional endpoint measurements to deeply reveal drug mechanisms of action, cell-type-specific responses, and resistance pathways (Ji et al., 2023). Integrated multi-omics pharmacodynamic evaluation helps identify early biomarkers, uncover unknown mechanisms and cell-type-specific reactions, support drug optimization, and identify resistant subpopulations and their adaptive metabolic reprogramming processes (Zhao et al., 2021). Several practical studies have validated the reliability of multi-organ chips in simulating toxic responses and metabolic pathway alterations. For example, Donald Ingber’s team—a pioneer in organ-on-chip technology—developed a highly integrated multi-organ chip system that coupled the functions of the liver, heart, lung, and other organs. Using scRNA-seq after drug intervention, they systematically monitored gene expression dynamics across different organ cell types, demonstrating the feasibility and advantage of such platforms for multi-omics mechanism research and efficacy evaluation (Ronaldson-Bouchard et al., 2022).

### 8.3 Technical challenges and standardization needs

Although organoids and organ-on-chip systems show great potential in modeling cardiac metabolic reprogramming, several key challenges remain for their practical application (Zhu et al., 2025). Current models have limitations in terms of cardiac cell maturity, structural organization, and functional stability, making it difficult to maintain adult metabolic phenotypes over the long term (Ren et al., 2025). Batch-to-batch variability and low reproducibility hinder standardized screening (Ronaldson-Bouchard et al., 2022), while high costs and limited throughput restrict large-scale drug screening applications (Probst et al., 2018). Addressing these challenges requires multi-faceted standardization and optimization. Examples include improving culture media and biomimetic materials to promote structural and functional maturation (Lewis-Israeli et al., 2021), establishing standard operating procedures (SOPs) and quality control measures to enhance consistency, introducing automated culture systems, microfluidic integration, and multi-modal detection to increase throughput and reduce costs, and conducting multi-center validation studies to clarify clinical predictive reliability (Yang et al., 2023). Regulatory agencies are actively promoting standardization in this field. For instance, the U.S. FDA has encouraged the use of organ-on-chip systems in cardiac safety assessment through related working groups, and China’s NMPA has also accepted organoid data as evidence of efficacy in guidelines (Zhou L. et al., 2025). Although globally unified standards have not yet been established, and issues such as batch variability and material adsorption remain unresolved, these developments indicate that such technologies are gradually being incorporated into regulatory frameworks, laying the foundation for future applications.

## 9 Challenges and future directions

### 9.1 Technical aspects

#### 9.1.1 Live-cell dynamic metabolic tracking technology

Studying cardiac metabolic reprogramming requires technologies capable of monitoring metabolic changes in living cells in real time. The highly dynamic and spatially heterogeneous nature of cardiac metabolism poses significant challenges for conventional detection methods. Förster resonance energy transfer (FRET)-based biosensors enable real-time monitoring of metabolic dynamics in live cells, providing a vital tool for dissecting cardiac metabolic reprogramming. This technique offers millisecond temporal resolution and subcellular spatial precision, allowing it to capture transient metabolite fluctuations and enzymatic activity changes without disrupting cellular integrity—a capability beyond the reach of traditional static omics approaches (Frei et al., 2024; Trumpp et al., 2025). It is particularly suitable for terminally differentiated cardiomyocytes and can reveal metabolic adaptations during stress, ischemia, and other pathological processes (Nayak et al., 2025; Chen et al., 2022). For example, the CUTie sensor, designed to detect localized cAMP signaling in the heart, revealed a near-complete loss of cAMP signals at TPNI sites during heart failure, suggesting that increased spatial heterogeneity may contribute to diastolic dysfunction (Divakaruni and Jastroch, 2022).

Although FRET has limitations, including photobleaching, uneven probe distribution, and quantitative calibration challenges, emerging technologies such as FRET-WGM microcavity sensing have significantly improved detection performance by enhancing signal sensitivity (Wang Y. et al., 2022). This technology offers a new tool for dynamically tracking cardiac metabolism. Future developments, including cardiac-specific FRET probes or highly biocompatible materials, could advance its application in cardiac electrophysiology and metabolic diseases. Next-generation FRET sensors are evolving toward multicolor encoding, expanded dynamic ranges, and near-infrared imaging (Hellweg et al., 2023). For instance, recently developed FRET sensors increasingly feature fully synthetic sensing modules. Through rational design and high-throughput screening to optimize response kinetics, these sensors substantially improve our ability to resolve cellular signaling networks in complex physiological environments (Zhao et al., 2023). Furthermore, combining FRET with techniques like mass spectrometry and microfluidics enables the simultaneous acquisition of dynamic and comprehensive data in controlled microenvironments, providing conditions closer to the *in vivo* setting for cardiac metabolic research (Rutkauskaite et al., 2022).

Beyond FRET, the Seahorse XF Analyzer provides insights into population-level energy metabolism phenotypes but cannot resolve single-cell heterogeneity (Divakaruni and Jastroch, 2022). Emerging techniques, such as spatial isotope tracing imaging and integrated microfluidics-mass spectrometry, allow researchers to track the fate and flux of labeled substrates, revealing metabolic heterogeneity and inter-organ communication (Li X. et al., 2025; Xu et al., 2024). Live-cell dynamic metabolic tracking addresses the gaps in temporal and spatial resolution inherent in traditional omics methods. It holds great potential, particularly for uncovering metabolic heterogeneity, subcellular compartmentalization, and the cross-talk between metabolism and signaling. Future efforts should focus on balancing detection throughput with resolution, and specificity with coverage, while also developing supporting bioinformatics tools. These advances are expected to enable the construction of a comprehensive, dynamic atlas of cardiac metabolism at the single-cell level, supporting the development of precise intervention strategies.

#### 9.1.2 Machine learning-driven multi-omics data integration

Single-cell multi-omics technologies provide vast amounts of data for analysising cardiac metabolic reprogramming, but the heterogeneity, high dimensionality, and non-linear interactions of cross-omics data pose challenges for integrated analysis. Machine learning (ML), deep learning, and graph neural networks have emerged as key technologies (Morabito et al., 2025). Through fusion learning and multi-modal alignment, they can reveal regulatory relationships between metabolic enzymes, transporters, and non-coding RNAs, enabling the construction of spatiotemporally specific metabolic networks (Argelaguet et al., 2021). For example, metabolic information neural networks (MINNs) integrate multi-omics data with genome-scale metabolic models (GEMs), improving the accuracy of flux predictions (Tazza et al., 2025). However, existing models still face challenges such as data noise, batch effects, and limited interpretability. Artificial intelligence methods are gradually addressing these issues, for instance, by using graph neural networks to build more comprehensive knowledge networks of metabolic reactions (Zhang et al., 2025). Future efforts should focus on developing semi-supervised learning frameworks that incorporate prior knowledge of metabolic pathways, integrate spatial transcriptomics and metabolic imaging data, and dynamically resolve metabolic hubs in cardiomyocytes under stress (Wang Y. et al., 2025). Additionally, the heterogeneity of multi-omics data makes integration difficult, necessitating unified data standards and quality control protocols, cross-platform integration algorithms, and benchmark datasets for validation (Simões and Riley, 2022). Particularly for cardiomyocytes, it is essential to consider the differences in transcriptomic features between single-cell and single-nucleus sequencing data (Paik et al., 2020).

### 9.2 Clinical translation

#### 9.2.1 Biomarker discovery: from single-cell data to circulating metabolite detection

Precision diagnosis and treatment of cardiac metabolic diseases represent a major frontier in modern cardiology. Advances in single-cell multi-omics technologies now allow researchers to resolve metabolic heterogeneity in cardiac cells with unprecedented resolution, facilitating the discovery of novel biomarkers and the development of precision-targeted therapeutic strategies. The following section focuses on the translation of biomarkers from single-cell data to circulating metabolite detection, as well as advanced delivery systems for metabolic modulators.

Single-cell multi-omics technologies provide powerful tools for discovering cardiac metabolic biomarkers. High-throughput analysis of cardiac cell subpopulations can identify metabolic enzymes and metabolites that are dysregulated in disease states and correlate them with circulating metabolite profiles. For example, scRNA-seq has revealed a shift from fatty acid oxidation to glycolysis in cardiomyocytes during heart failure, with changes in key enzymes such as CPT1B and PDK4 affecting circulating metabolite levels (e.g., lactate, acylcarnitines). These metabolites act as signaling molecules regulating epigenetic modifications and cellular function (Kliesow Remes et al., 2025; Hua et al., 2025). Additionally, diagnostic value of gene signatures related to heart failure with reduced ejection fraction (HFrEF) in peripheral blood is comparable to that of BNP(225). The circulating tumor cell metabolomics technology developed by Qi Wang’s team also offers insights for cardiac metabolic research (Xu et al., 2025). Recent studies have identified various circulating metabolites associated with cardiac metabolic diseases, such as imidazole propionate (ImP) and trimethylamine-N-oxide (TMAO), which promote atherosclerosis by modulating inflammation and lipid metabolism (Mastrangelo et al., 2025; Li et al., 2021). Long-chain acylcarnitines are key intermediate metabolites in fatty acid oxidation (Wu Z. et al., 2025). Their accumulation reflects impaired mitochondrial function and exhibits cytotoxicity (Liepinsh et al., 2016; Xie H. et al., 2023). Studies have shown that decreased levels of long-chain acylcarnitines (e.g., C16) may indicate improved cardiac metabolism and function (Baskhanova et al., 2025). Machine learning methods, such as BioMapAI, integrate multi-omics data to accurately classify disease states and identify biomarker combinations, further accelerating biomarker discovery (Xiong et al., 2025). In cardiovascular research, similar approaches may reveal new diagnostic biomarker panels (e.g., APOA/HPT combination) and drug targets (e.g., IGF1R/RAF1) (Lin M. et al., 2025).

Clinical translation requires rigorous validation. Prospective studies have shown that metabolomic-based models can provide early warnings of metastatic events; similar strategies could be applied to predict heart failure risk (Xu et al., 2025). Multi-marker panels often exhibit superior performance, and dynamic changes in circulating metabolites can serve as early indicators of treatment response. For example, SGLT2 inhibitor therapy alters blood levels of ketone body β-hydroxybutyrate and fatty acids, changes that correlate with improved cardiac function (Xu et al., 2025). Future approaches may involve guiding treatment selection based on metabolic subtypes, such as PFKFB3 inhibitors for glycolysis-dominant heart failure patients or CPT1 agonists for those with lipid metabolism abnormalities (Forte et al., 2024).

Circulating metabolite profiles indirectly reflect intracellular metabolic events in the heart, offering novel biomarkers for cardiovascular diseases and advancing precision medicine in diagnosis and treatment.

#### 9.2.2 Delivery systems for metabolic modulators

Efficient targeted delivery systems are essential for the clinical translation of metabolic modulators. Targeted delivery systems are critical for enhancing the efficacy of metabolic modulators and reducing side effects but face limitations due to cardiac cell heterogeneity and anatomical barriers. Current research focuses on delivering metabolic modulators (e.g., AMPK activators, mitochondrial antioxidants) specifically to certain cardiac cell types, such as cardiomyocytes, endothelial cells, or fibroblasts. Promising delivery systems include nanotechnology-based targeted delivery systems and molecularly engineered extracellular vesicles (EVs).

First, nanotechnology offers important solutions. For example, Peng Teng’s team developed lipid nanoparticles (LNPs) coated with platelet membranes to enhance cardiac targeting, delivering siRNA to improve myocardial hypertrophy (Teng et al., 2025). Polymer nanoparticles have been used to reprogram macrophages for treating myocardial fibrosis (Liu Z. et al., 2025). Biomimetic nanocarriers use cell membrane coating technology to improve targeting and biocompatibility, enabling precise enrichment in diseased areas (Cassani et al., 2020). For instance, gold nanocages loaded with metabolic modulators and modified with cardiac-targeting peptides have been shown to improve cardiac function (Wang et al., 2023c). Extracellular vesicles (EVs), as endogenous carriers, offer low immunogenicity and natural targeting abilities. Cardiac-derived EVs are rich in protective miRNAs and can mitigate injury by regulating signaling pathways; surface engineering can further enhance their targeting and circulation half-life. However, clinical translation faces challenges in large-scale production and standardization (Wang H. et al., 2025).

Several targeted delivery strategies have shown significant effects in disease models, such as silencing METTL3 to improve cardiac function and engineered CAR-MΦ to reduce fibrosis (Teng et al., 2025; Liu Z. et al., 2025). Additionally, multiple therapies have entered clinical research stages, including EVs for myocardial infarction (Wang H. et al., 2025); LNP-based RNAi therapies are expanding into cardiovascular applications, with siRNA targeting PCSK9 already successfully used for lipid-lowering treatment (Teng et al., 2025); CAR-MΦ therapy shows great potential against fibrosis by specifically clearing activated CFs, and the first clinical study evaluating CAR-MΦ for post-myocardial infarction fibrosis is in preparation, with patient recruitment expected to begin in 2026 (Liu Z. et al., 2025).

### 9.3 Challenges and future directions

Biomarker discovery and targeted delivery face multiple technical challenges. First, single-cell metabolomics has limited sensitivity and coverage, requiring standardized protocols and reference databases to improve comparability across laboratories (Zhang W. et al., 2023). The high heterogeneity of cardiac cells demands precise targeting; spatial multi-omics technologies (e.g., MERFISH, Seq-Scope) can guide the design of nanocarriers (Farah et al., 2024; Kim et al., 2025). Physical targeting (e.g., ultrasound-mediated drug release) and multi-stage targeting strategies (e.g., first targeting the heart, then specific cell types) may help address off-target effects and delivery efficiency issues (Wang H. et al., 2025).

It is important to note that advancing biomarkers and targeted therapies to the clinic requires solving many key issues. Large-scale, standardized production of extracellular vesicles and nanocarriers remains a bottleneck for clinical translation. Future efforts should develop microfluidic synthesis platforms and continuous production processes to improve yield and quality control. Additionally, long-term safety evaluation systems for metabolic modulators need to be established, particularly for systematic assessment of systemic effects resulting from interference with metabolic networks. Regulatory agencies must develop review guidelines adapted to the characteristics of metabolic-targeted therapies, including co-approval pathways for companion diagnostics and evaluation frameworks for personalized treatments.

Furthermore, future cardiac metabolic therapies will increasingly emphasize personalized strategies. Patients could be stratified into subtypes based on single-cell metabolic profiles to guide treatment selection. For example, glycolysis-dominant heart failure patients may benefit from PFKFB3 inhibitors, while those with lipid metabolism abnormalities may be more suitable for CPT1 agonists. Continuous monitoring of circulating metabolites can assess treatment response and allow dynamic adjustment of therapeutic regimens. Integrating wearable metabolite sensors with artificial intelligence algorithms could enable real-time treatment optimization. For multiple metabolic abnormalities, combination therapies may be employed. For instance, combining SGLT2 inhibitors (improving glucose metabolism) with carnitine supplements (enhancing fatty acid oxidation) may synergistically improve cardiac energy metabolism.

Single-cell multi-omics is driving a shift toward precision diagnosis and treatment in cardiac metabolic diseases. Integrating single-cell data with circulating metabolite analysis can identify specific biomarkers, while nanotechnology and bioengineering enable precise delivery. Future efforts should focus on multi-omics integration, metabolic network analysis, and delivery system optimization to advance personalized medicine. With the refinement of clinical trials and regulatory pathways, biomarker-guided targeted therapies are expected to become routine strategies, improving patient outcomes.

## 10 Discussion

Single-cell multi-omics technologies have fundamentally reshaped our understanding of cardiac metabolism, shifting from tissue-level averaged profiles toward precise, cell type- and even subpopulation-specific insights. These studies have established metabolic reprogramming as a driver, rather than a bystander, of disease progression. They have uncovered novel intercellular communication mechanisms such as lactate shuttling and lipid metabolite exchange, and identified repair-associated cell subpopulations, for example, GATA5/ISL1 positive fibroblasts, thereby opening new avenues for targeted therapies. However, translating these discoveries into the clinic remains challenging due to technical limitations, such as low cardiomyocyte capture efficiency and the complexity of multi-omics integration, along with biological redundancy and a lack of clinical-grade validation. We anticipate that biomarkers and metabolic interventions based on single-cell profiling may enter clinical evaluation within the next 5–10 years. Yet, achieving precision medicine will require deep integration of computational biology, metabolic science, and clinical cardiology, along with interdisciplinary efforts to overcome translational barriers in technology innovation, target validation, and clinical trial design. Ultimately, these efforts will transform mechanistic insights into effective therapies that improve patient outcomes.
